# What compels enrollment in a mobile maternal health wallet? A mixed-methods doer/non-doer analysis in Analamanga, Madagascar

**DOI:** 10.1186/s12913-025-13770-x

**Published:** 2025-12-06

**Authors:** Louis Noël Schäfer, Zavaniarivo Rampanjato, Lisa Bogler, Elsa Rajemison, Samuel Knauss, Julius Valentin Emmrich, Mirana Larissa Randriamiarisoa, Harizaka Emmanuel Andriamasy, Louisa Marie Truss, Bítia Vieira, Till Bärnighausen, Mark Donald C. Reñosa, Shannon A. McMahon

**Affiliations:** 1 Heidelberg Institute of Global Health, Medical Faculty and University Hospital, University of Heidelberg, Heidelberg, Germany; 2 Global Digital Last Mile Health Research Lab, Charité Center for Global Health, Charité - Universitätsmedizin Berlin, Berlin, Germany; 3 Ministry of Public Health of the Republic of Madagascar, Antananarivo, Madagascar; 4 Department of Economics and Centre for Modern Indian Studies, Georg-August-Universität Göttingen, Göttingen, Germany; 5 Department of Neurology, Charité - Universitätsmedizin Berlin, Berlin, Germany; 6 Berlin Institute of Health, Berlin, Germany; 7 Doctors for Madagascar, Antananarivo, Madagascar; 8 Department of Global Health and Population, Harvard T. H. Chan School of Public Health, Harvard University, Boston, MA USA; 9 Africa Health Research Institute (AHRI), Somkhele and Durban, South Africa; 10 Department of Epidemiology and Biostatistics, Research Institute for Tropical Medicine, Department of Health, Muntinlupa, Philippines; 11 Johns Hopkins Bloomberg School of Public Health, Baltimore, MD USA

**Keywords:** MHealth, Health financing, Maternal health, Health wallet, Mobile money, Doer/non-doer analysis, Madagascar, Mixed methods

## Abstract

**Background:**

Care-seeking during antenatal and intrapartum periods has remained persistently low in Madagascar, largely due to financial barriers. To bolster both care-seeking and health financing, a Mobile Maternal Health Wallet (MMHW) was developed and implemented in the Analamanga region, Madagascar. The MMHW, a service based on mobile money, enabled users to digitally save and pay for services at participating public health facilities. Here, we compare perspectives of those who enrolled (doers) and those who did not enroll (non-doers) in the MMHW to understand decision-making processes, interpersonal dynamics, and other factors that informed enrollment.

**Methods:**

In this mixed methods study, we analyzed data from a quantitative survey (*n* = 477) examining predictors of enrollment using logistic probability models, followed by applying Reflexive Thematic Analysis to qualitative in-depth interviews (*n* = 29) to gain insights from 11 doers, 12 non-doers, three family members, and three MMHW outreach team members.

**Results:**

Quantitatively, significant predictors of enrollment included: learning about the MMHW from a midwife, having a pre-existing maternal medical risk factor, and residing in the Avaradrano district of Antananarivo. Predictors of non-enrollment included: learning about the MMHW from family or friends, having a higher salary, and being diagnosed with medical warning signs during pregnancy. Qualitative findings mostly mirrored the quantitative data, revealing that women enrolled due to early, comprehensive information from trusted sources (e.g., midwives) and because they found the offer of financial benefits and expanded medical services meaningful and reliable. Women sometimes reported that peer influence played a role as driver of enrollment, though not uniformly. Women who were non-doers described a sense of incredulity about the MMHW (often citing rumors or others’ negative experiences), incomplete information, spousal disagreements about enrolling, and implementation-related barriers (e.g., lacking an ID card, which was needed for enrollment).

**Conclusion:**

This study underscores the importance of evaluating sensitization activities among target populations. Our findings highlight a critical need to identify trusted information sources (individuals and channels) and to convey innovative programs via these sources. Effective communication and the elimination of implementation barriers remain essential to bolster care-seeking and improve maternal health outcomes in Madagascar and beyond.

**Trial registration:**

This study is a component of the 4MOTHERS trial, which was registered on March 12, 2021, in the German Clinical Trials Register (DRKS), identifier: DRKS00014928, https://drks.de/search/en/trial/DRKS00014928.

**Supplementary Information:**

The online version contains supplementary material available at 10.1186/s12913-025-13770-x.

## Introduction

Financial obstacles hinder maternal healthcare globally [1–4]. In Madagascar, widespread poverty coupled with high out-of-pocket costs (96.5% of Malagasy women lack health insurance) [5–7] underpin a system where: 40% of women complete less than four antenatal visits (data related to more than four antenatal care visits are currently unavailable) [6]; rates of facility-based childbirth have stagnated at ~ 40% for decades [6]; and the maternal mortality rate remains high, with 445 deaths per 100,000 live births [7].

In several sub-Saharan countries, including Madagascar, interventions involving the elimination of user fees have reduced maternal mortality and increased healthcare uptake [8–10]. In Madagascar, however, fee reductions were not expanded or have since been eliminated, with costs and sustainability challenges cited as reasons for discontinuation [8, 11]. Other strategies throughout Africa to support the healthcare system while avoiding catastrophic healthcare expenditures for clients have entailed targeted insurance programs (in Ghana [12] and Nigeria [13]); community-based health insurance (CBHI) schemes (in Senegal, Mali, and Ghana [14]); cash transfer programs (in Uganda, Kenya, and Nigeria [15]), and voucher schemes (in Cameroon, Tanzania, Kenya, Uganda, and Madagascar [16–18]).

Developing health finance mechanisms that employ technologies such as mHealth (mobile health) and mobile money, including mobile health wallets, is a relatively recent approach to improving health financing [19–21]. The aim of mobile health wallets, which operate via smartphones and feature phones, is to enable financial savings that could subsequently facilitate access to healthcare. Implementation research in Kenya, Rwanda, Tanzania, and Madagascar has described opportunities and challenges related to the implementation of mobile health wallets, focusing on the perspectives of those who enroll in such programs [22–24]. Relatively few studies examine why individuals do not enroll in an intervention, particularly if they were sensitized to the intervention, and if the intervention was expressly designed to address their needs as, for example, a high-risk or low-income patient.

An approach to knowledge generation that entails active engagement with those who have not enrolled in an intervention is referred to as a “doer/non-doer analysis” [25] or “barrier analysis” [26, 27] and can entail quantitative [28–30], qualitative [31–33] or mixed methods approaches [34]. We are not aware of such analyses applied to maternal health financing projects generally or maternal health wallets specifically.

Two formative studies preceded the implementation of the MMHW and guided the research team to consider how low phone use, illiteracy and costs may pose challenges for uptake, whereas a user-friendly design, clear information, local collaboration, and incentives to save could facilitate uptake [35, 36]. The impact of the MMHW on maternal and neonatal health outcomes was analyzed elsewhere [37]. This doer/non-doer analysis contributes to understanding factors that ultimately influenced eligible women and their families to decide for or against enrollment in the program.

## Methods

### Study setting

We conducted this study in three districts constituting and surrounding Madagascar’s capital, Antananarivo (Atsimondrano, Renivohitra, and Avaradrano) within the country’s Analamanga region, including urban and semi-urban/rural areas [38] (for a detailed map, visualizing the implementation area see Lacroze et al. 2021 [39]). Certain basic services concerning antenatal care and birth are free within the public health system of Madagascar (all ANC visits including counselling, examinations, basic diagnostics, and hospital stays), but pregnant women and their families typically must pay for medication (also during hospital stays), ultrasounds, ambulance transport, surgery (including cesarian sections), and other services and procedures [8, 40]. In terms of antenatal care specifically, eight visits were recommended by the WHO and the Malagasy Ministry of Public Health when the study was conducted [41, 42]. As in many countries, Antananarivo, as the capital, has a higher rate of health facility-based births than the national average (68.7% vs. 38.5%) [6]. Non-public providers of medical services related to pregnancy and birth include private medical practices and hospitals, and traditional birth attendants [43, 44].

Mobile money is widely used in Madagascar and active accounts more than quadrupled between 2016 and 2022 [45]. As of 2021, 34.6% of women aged 15–49 in Madagascar owned a mobile phone and 56.4% of these women used it for financial transactions, while in the Analamanga region, 53.0% of women owned a mobile phone and 53.5% of them used it for financial transactions [6].

### Intervention design

The MMHW was developed as part of a trial that began in 2019 and is referred to as “4MOTHERS” or the “Madagascar Mobile Money for Maternal Healthcare-Related Spending” trial. The MMHW was implemented in 29 public basic health facilities (CSBs, centres de santé de base, which offer basic primary and obstetric healthcare services) as well as four larger referral hospitals (which provide a broader spectrum of services allowing diagnostics and treatment for high-risk cases referred from CSBs). The program entailed outreach agents and healthcare providers sensitizing women on the wallet during the antenatal period. Mass sensitization events also occurred in communities in partnership with community health workers. Furthermore, women were approached by *reny mavitrika*, women who had used the MMHW themselves and shared their experiences. Eligible women could register at health facilities or sensitization events. For enrollment, a national ID card and a photo were required. After enrollment, the woman received a SIM card that she could use in any phone she had access to (either her own or somebody else’s phone). The saving and payment platform was operated with the software *mTOMADY* using Unstructured Supplementary Service Data (USSD). Users could access services with smartphones or feature phones as the services offered did not require internet access. Depositing money was possible at any mobile money cash point of the respective mobile money provider as well as at participating public health facilities [24]. If families used their savings to pay for maternal health services at a participating facility, an implementation partner, *Doctors for Madagascar*, matched the savings with a 50% bonus. The wallet further entailed vouchers for free ultrasound examinations, selected medication (Iron, Albendazole), and ambulance transportation in case of complications [39].

### Study design and data integration

This study applied a mixed methods approach [46]. As we observed amid quantitative data collection that a meaningful proportion of surveyed women who were eligible and had heard about the MMHW had not enrolled, we actively sought out doers and non-doers for qualitative interviews. Based on findings from the qualitative interviews, we developed hypotheses regarding relevant topics that could inform quantitative analysis. We ultimately analyzed both datasets fully, comparing results from quantitative and qualitative streams to inform an integrated analysis. For data reporting, we followed the STROBE guideline for quantitative data and the COREQ guideline for qualitative data [47, 48]; information required by a guideline but not specified in this article can be found in Supplementary file 1.

### Quantitative data

#### Study design and sampling

Quantitative data were derived from the 4MOTHERS randomized controlled trial household survey (see protocol [39]). Data were collected between February and December 2022 via a face-to-face survey with women (aged >18 years) who had completed their pregnancy between July 1, 2020, and December 31, 2021. A total of 6,483 women completed this survey.

We used a subset of the quantitative household data to analyze the association between enrolling in the MMHW and a set of individual characteristics. We included a woman in the analysis if (a) she or her household had to pay out-of-pocket for not publicly covered healthcare expenses, i.e. she had no health insurance nor was enrolled in another NGO-sponsored program, (b) she went to a health facility where the MMHW was implemented for at least one antenatal care visit, (c) she gave birth in a facility where the MMHW was implemented, (d) she had heard about the MMHW and could explain what it is. We chose these criteria to include women who could form an opinion on the MMHW and describe reasons for (non-)enrollment. The criteria led to a sample size of 689 women. Low rates of women who had heard of the MMHW, even in the intervention area, led to a sample that is notably smaller than the full sample of the randomized controlled trial.

#### Statistical analysis

We estimated logistic probability models with robust standard errors to identify characteristics associated with MMHW enrollment.

The dependent variable is a dummy equal to 1 if the individual enrolled in the MMHW, meaning she is a “doer”, as opposed to not having enrolled in the MMHW, i.e., a “non-doer”. As covariates, we included a set of individual characteristics, informed by findings from the qualitative interviews: maternal age (18–24 years, 25–34 years, 35–55 years), marital status (married/not married), number of children, education level (no or primary, secondary, or higher education), access to a mobile phone (yes/no), monthly salary, maternal medical warning signs during pregnancy (yes/no; e.g., edema, glucosuria, vaginal bleeding), having a pre-existing maternal medical risk factor (yes/no; e.g., age > 35 years, multiple pregnancy, hypertension), maternal involvement in household decision-making related to finances (yes/no), source of information regarding the MMHW (e.g., family or friends, midwife, other), district of residence.

The multivariate regression analysis contains only those observations with complete information on all covariates included in any of the specifications, leading to an analysis sample size of 477. A flowchart depicting the cases lost due to missing information can be found in Supplementary file 3.

### Qualitative data

#### Study design and sampling

**Fig. 1:**
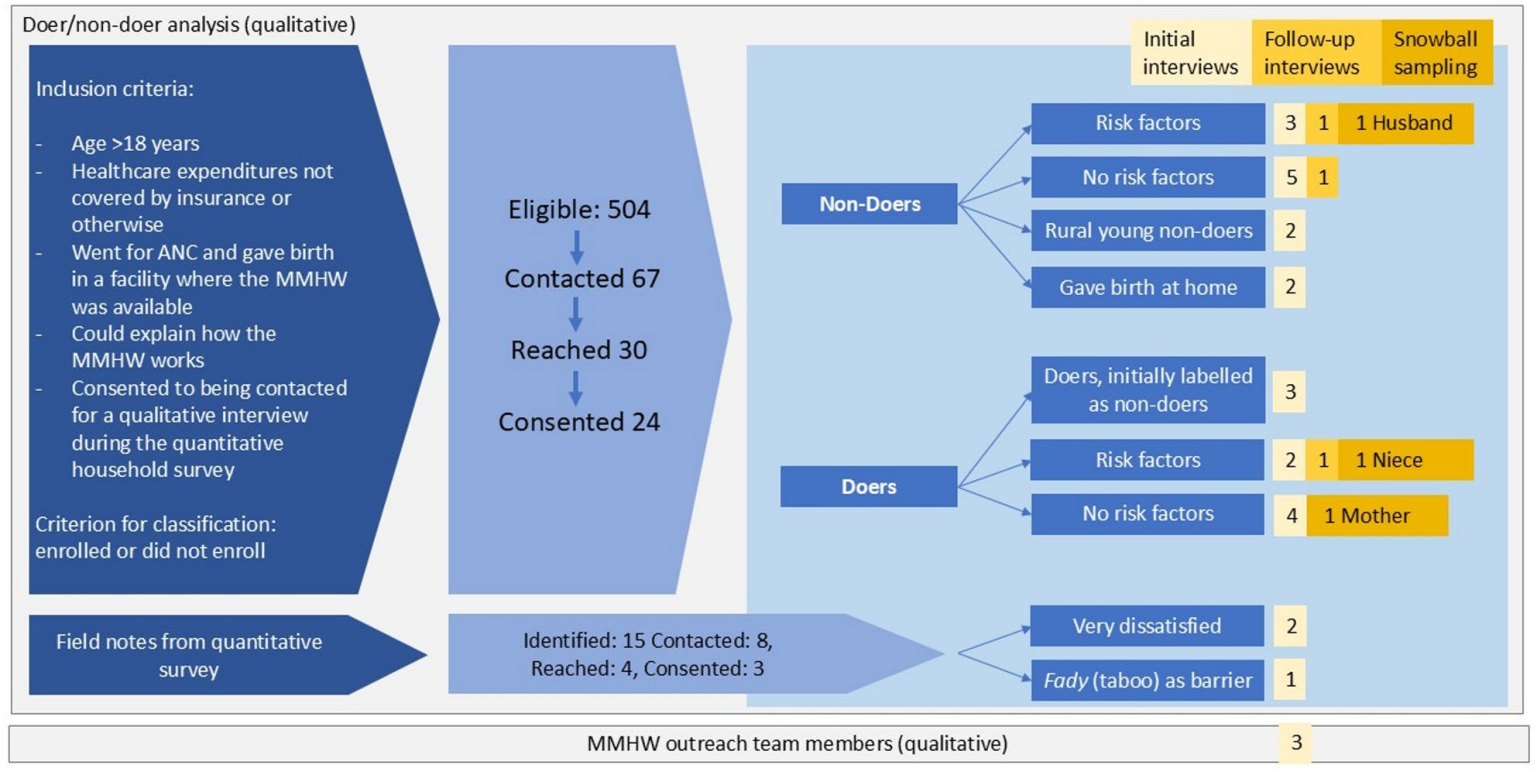
Eligibility criteria and sampling strategy for qualitative interviews

The qualitative team identified doers (MMHW-enrolled) and non-doers (non-enrolled) with and without pre-existing maternal medical risk factors (see Fig. 1). We followed a purposive sampling approach and included respondents from a variety of district types (urban vs. semi-urban/rural), age, education, and marital status. Snowball sampling was applied to approach other family and community members identified as critical cases. A main barrier to contacting respondents was the absence of phone numbers; a main reported reason for interview refusal was a lack of time. During interviews, we categorized three respondents originally identified as non-doers (based on quantitative data) as doers. Two of these respondents used the wallet for a previous pregnancy but not for the most recent pregnancy (due to giving birth in a facility where the MMHW was unavailable in one case and being unaware of a pregnancy due to other health conditions in another case). In a third case, a woman enrolled but later discontinued.

#### Data collection

Two research assistants with previous experience in qualitative data collection and fluent in Malagasy and French underwent a two-day training and four half-day refreshers on the foundations of qualitative research, interview techniques, and research ethics. Interview guides for in-depth interviews for doers and non-doers differed slightly, but both included the following topics: MMHW first impressions, household decision-making on the MMHW and healthcare seeking, perspectives on saving money, mobile technology, and mobile money as well as peers’ experiences with the MMHW (see Supplementary file 2). The interview guide for doers additionally entailed questions about the individual experiences with the MMHW. Based on initial guides, the research team developed additional interview guides for other family members. The interview guides for outreach team members covered topics concerning the MMHW implementation process and themes that were identified during the interviews with doers and non-doers. All interview guides were translated into Malagasy by a professional translator and translated back into English by a research team member. Interview guides were pilot tested with two respondents and refined accordingly.

Qualitative data collection took place from August to November 2022. Respondents were contacted two to seven days in advance by phone. The research team explained the qualitative study’s aims and conditions and scheduled an interview appointment. Interviews (range: 18–91 min) were conducted in a quiet place as chosen by respondents. Data were transcribed verbatim by the interviewers and translated into English by professional translators. ZR and ER performed quality checks on transcription and translation. One interview was later removed from the dataset because although the respondent met the inclusion criteria, during an exceptionally short interview she declined to respond to most questions, citing a lack of knowledge and interest.

We undertook systematic debriefings after each interview. Debriefings improved later interviews, informed the sampling strategy, facilitated the identification of topics, and supported data analysis [49]. One senior author (SAM) debriefed the qualitative core research team (LNS, ZR, ER) weekly. We terminated data collection once saturation for all groups was reached.

#### Data analysis

We analyzed the transcripts guided by Reflexive Thematic Analysis [50]. When all data were collected, the lead author (LNS) inductively coded six information-rich interview transcripts to develop a preliminary codebook. ZR and MDCR reviewed the initial codebook and suggested adding more sub-themes and removing coding overlaps, which they discussed with LNS. After adopting the suggested changes to the codebook, LNS coded all interview transcripts using NVivo 12, continuously amending and refining the codebook. ZR coded three Malagasy transcripts to check if the coding aligned with LNS. The research team also used notes from qualitative interviews and debriefings to triangulate data and critically discussed the preliminary results regularly.

## Results

### Sample characteristics

The quantitative dataset included 267 doers and 210 non-doers (see Table 1). The qualitative dataset included 11 doers and 12 non-doers; additionally, interviews were conducted with three family members and three MMHW outreach team members. Most mothers in both samples were between 18 and 24 years old, married, and had one or two children. Most were self-employed or unemployed and lacked regular income. Approximately half of mothers were living in urban areas, and around half of mothers reported medical risk factors.

**Table 1:** Sample characteristics

All respondents		Quantitative		Qualitative
		*n*	%	*n*
Respondent group	Mothers – doers	267	55.97	11*
	Mothers – non-doers	210	44.03	12
	Family members	-	-	3
	MMHW outreach team members	-	-	3
Gender	Female	477	100.00	28
	Male	-	-	1
**Mothers**				
Age	18–24	215	45.07	13
	25–34	199	41.72	8
	35–55	63	13.21	2
Marital status	Married	435	91.19	21
	Not married	42	8.81	2
Number of children	0	1	0.21	-
	1	174	36.48	11
	2	145	30.40	6
	3	91	19.08	3
	4	40	8.39	1
	5 or more	26	5.45	2
Occupation**	Wage earner	19	3.98	-
	Self-employed	187	39.20	11
	Family farm / livestock / fishing	69	14.47	-
	Student	3	0.63	-
	Not working	199	41.72	12
School level	No education	6	1.26	-
	Primary education	127	26.62	6
	Secondary education	303	63.52	14
	Higher education	41	8.60	3
Regular income	Yes	216	45.47	8
	No	259	54.53	15
Salary last 30 days***	< 100,000 Ar (< $ 23.5)	133	27.88	6
	100,000–199,999 Ar ($ 23.5 – $ 47)	118	24.74	3
	200,000–399,999 Ar ($ 47 – $ 94.5)	165	34.59	5
	>=400,000 Ar (>$ 94.5)	61	12.79	2
	No data	0	0	7
Residence	Urban area	214	44.86	13
	Semi-urban / rural area	263	55.14	10
Pre-existing maternal medical risk factor	Yes (e.g., age < 18 or > 35, primipara > 30, multiple pregnancy, hypertension, retracted or asymmetric pelvis, any uterus surgery, history of low birth weight, toxic habit, history of miscarriage/abortion)	229	48.01	9
	None	248	51.99	14

* Including doers initially labelled as non-doers

** Multiple choice possible in quantitative survey

*** Exchange rates Ariary/US-Dollar as of October 1st, 2022

### Who enrolls, and who does not?

**Fig. 2:**
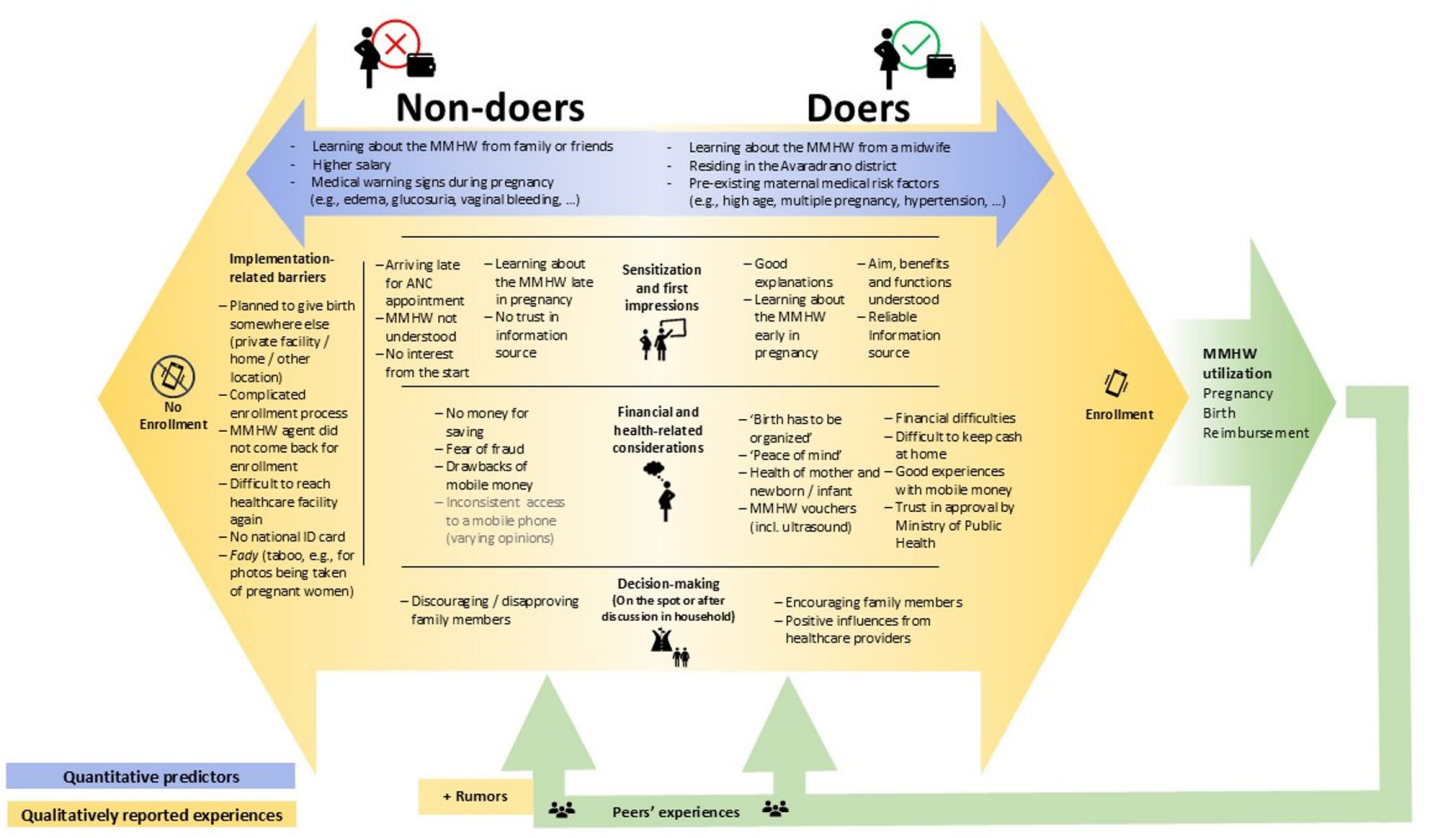
Quantitative predictors and qualitatively reported factors influencing enrollment or non-enrollment in the Mobile Maternal Health Wallet (MMHW)

An overview of quantitative predictors and qualitatively reported factors that underpin enrollment, or non-enrollment is available in Fig. 2. Additional quotes supporting the statements made in the results section can be found in in Table 2, the original quotes from the qualitative interviews in Malagasy cited in this article in Supplementary file 4.

**Table 2:** Additional qualitative quotes

Theme	Supporting quote
Compelled by explanations at a CSB	*“It was at the CSB […]. There*,* an MMHW agent explained what the MMHW is and said that it can help you. If you have a problem during childbirth*,* the MMHW can help you. Especially with the money. As we are in need*,* we were very motivated*,* because in case of complications*,* the MMHW would help.”* (doer, aged 30, 1 child)
Not asking questions during MMHW sensitization due to shyness and discomfort	*“[The MMHW agent] told me to ask questions if I had any and my question was: ‘If I don’t have any money to put in the SIM-card [laughs]*,* what will happen?’ And I didn’t ask the question because it was only me who had the question and the others didn’t ask as well!”* (non-doer, aged 34, 5 children)
Not understanding the MMHW’s benefits or premise	*“I don’t see the advantage because it’s the same. We always must pay money. […] I do not see any disadvantage; it is the same whether we enroll or not*.” (non-doer, aged 23, 2 children)
Approached too late to benefit	*“First*,* I didn’t intend to enroll. Why? Because two months before I gave birth*,* I met [the MMHW agent]! Because I usually did the prenatal examination [in a CSB] and sometimes [the agent] was there and sometimes not […] And I wasn’t interested anymore because no… I thought I ‘m already about to give birth and I don’t really need it!”* (non-doer, aged 34, 5 children)
Community conversations on the MMHW	*“The way they talk is divided in two. Some […] say: don’t enroll because it does not make sense. The others encourage you to use the MMHW because as they used it*,* the delivery was lighter for them. They did not spend a lot of money.”* (doer, aged 38, 4 children)
Sharing personal experiences about the MMHW with others	*“When I meet some of the pregnant women here*,* I advise them to [enroll in the MMHW] because it is really great to be a member*. [...] *I explain to them what we can get from the MMHW.”* (doer, aged 30, 1 child)
Having no money to save	*“We never save because we are in need. If there is an emergency*,* we have to borrow. […] Because you spend what you earn and you cannot block the money there when you have nothing to eat.”* (non-doer, aged 22, 2 children)
Husband harboring doubts about an MMHW information source	*“Personally*,* first of all the question of scamming for example. The question of payment made me think: Who are you going to give the money to? Where? Who is going to check it? […] it was just a conversation in the street with the midwife. The truth is that there are many scammers.”* (husband of a non-doer, aged 25)
Wife-husband pairs jointly making decisions	Interviewer: “*Who discusses [health issues in the family]?*” Respondent: “*My husband and I*.” Interviewer: “*And who decides to seek treatment?*” Respondent: “*We decide together.*” (non-doer, aged 22, 2 children)
Husbands being described as primary decision makers	*“As I said*,* it is me and my husband who discuss but he often decides.“* (non-doer, aged 22, 1 child)
Enrollment process perceived as too complex	*“I thought about enrolling but I was still struggling because I was pregnant and then there were several steps to enroll.”* (non-doer, aged 28, 1 child)
Prior familiarity with mobile technology and mobile money facilitating MMHW enrollment	*“Yes*,* using the phone is quite easy. The obstacle that exists is that some people don’t know how to handle a phone and would have to go to a CSB to do so.”* (non-doer, aged 20, 2 children)
Owning a phone described as not crucial	*“We approached [the MMHW agent] because my daughter was ready to [enroll]*,* she brought her ID card there. Whether or not she did her prenatal consultation*,* she came there to deposit money. Even if it is not a lot of money*,* they said*,* you could always deposit even 1*,*000 ariary. There is a great advantage because when the day arrived*,* we were in a period of nothing. We only took the SIM card and the identity card with us*,* she gave birth at the CSB […]*,* and we paid the expenses with [the MMHW].”* (mother of a doer, aged 42)

### Quantitative predictors

Quantitatively, learning about the MMHW from a midwife (model 3 OR = 1.667; 95%CI = 1.0588–2.6245), having a pre-existing maternal medical risk factor (model 4 OR = 1.531; 95%CI = 1.0269–2.2833), and residing in the Avaradrano district of Antananarivo [Avaradrano district (rather semi-urban/rural) compared to Atsimondrano district (rather semi-urban/rural) in model 4 OR = 2.891; 95%CI = 1.6082–5.1975; Renivohitra district (rather urban) compared to Atsimondrano district in model 4 OR = 1.112; 95%CI = 0.7338–1.6854], were positively associated with being a doer. To further investigate the influence of geographical location, we performed a robustness check on the quantitative data, adding an indicator for urban/rural as a covariate (based on *fokontany*, smaller administrative units than districts). However, the indicator did not show any association with being a doer or non-doer.

Learning about the MMHW from family or friends (model 2 OR = 0.433; 95%CI = 0.2047–0.9143), having a higher salary (model 4 OR = 0.884; 95%CI = 0.7875–0.9914), as well as being diagnosed with maternal medical warning signs that may indicate potentially serious complications during pregnancy (e.g., edema, glucosuria, vaginal bleeding) (in models 1 and 3; model 1 OR = 0.436; 95%CI = 0.1975–0.9614; model 3 OR = 0.442; 95%CI = 0.1969–0.9931; model 4 OR = 0.454; 95%CI = 0.1981–1.0399), were associated with being a non-doer. We found no statistically significant associations between being a doer or non-doer and women’s involvement in decisions on household finances, phone ownership or sociodemographic variables such as age, number of children, marital status, and education level (see Table 3).

**Table 3:** Quantitative associations between chosen variables and enrollment

	Model 1 Not including information sources	Model 2 Including midwife as information source on the MMHW	Model 3 Including family or friends as information source on the MMHW	Model 4 Including midwife and family or friends as information source on the MMHW
Age 17–24	Ref.	Ref.	Ref.	Ref.
Age 25–34	1.188	1.230	1.194	1.227
	(0.7385–1.9121)	(0.7628–1.9838)	(0.7390–1.9276)	(0.7596–1.9822)
Age 35–55	1.168	1.243	1.167	1.228
	(0.5059–2.6970)	(0.5313–2.9082)	(0.4977–2.7383)	(0.5201–2.8997)
Married	1.352	1.417	1.366	1.411
	(0.6805–2.6845)	(0.7156–2.8056)	(0.6806–2.7403)	(0.7064–2.8192)
Number of children	0.953	0.941	0.936	0.930
	(0.7764–1.1696)	(0.7659–1.1555)	(0.7598–1.1540)	(0.7552–1.1459)
No / Primary education	Ref.	Ref.	Ref.	Ref.
Secondary education	1.180	1.158	1.162	1.150
	(0.7652–1.8192)	(0.7498–1.7887)	(0.7508–1.7985)	(0.7430–1.7791)
Higher education	0.917	0.851	0.865	0.826
	(0.3867–2.1735)	(0.3560–2.0323)	(0.3612–2.0704)	(0.3440–1.9809)
Mobile phone ownership	1.198	1.187	1.190	1.185
	(0.8028–1.7880)	(0.7949–1.7731)	(0.7961–1.7802)	(0.7925–1.7714)
Salary in the last 30 days (in 100,000 Ariary)	0.868*	0.880*	0.877*	0.884*
	**(0.7743–0.9728)**	**(0.7830–0.9885)**	**(0.7825–0.9819)**	**(0. 7875–0.9914)**
Maternal medical warning signs during pregnancy	0.436*	0.455	0.442*	0.454
	**(0.1975–0.9614)**	(0.2013–1.0280)	**(0.1969–0.9931)**	(0.1981–1.0399)
Pre-existing maternal medical risk factors	1.518*	1.529*	1.523*	1.531*
	**(1.0213–2.2562)**	**(1.0265–2.2773)**	**(1.0227–2.2675)**	**(1.0269–2.2833)**
Woman involved in decisions on household finances	1.501	1.409	1.386	1.343
	(0.8299–2.7144)	(0.7797–2.5473)	(0.7709–2.4934)	(0.7450–2.4212)
Learned about the MMHW from family or friends		**0.433** ^*^		0.518
		**(0.2047–0.9143)**	**(1.0588–2.6245)**	(0.2385–1.1259)
Learned about the MMHW from a midwife			**1.667** ^*^	1.499
			**(1.0588–2.6245)**	(0.9396–2.3927)
Atsimondrano District	Ref.	Ref.	Ref.	Ref.
Renivohitra District	1.126	1.123	1.113	1.112
	(0.7461–1.7000)	(0.7413–1.7001)	(0.7357–1.6832)	(0.7338–1.6854)
Avaradrano District	2.825***	2.997***	2.740***	2.891***
	**(1.5803–5.0487)**	**(1.6688–5.3821)**	**(1.5336–4.8945)**	**(1.6082–5.1975)**
Observations	477	477	477	477
Pseudo-R^2^	0.0531	0.0601	0.0604	0.0643

Odds ratios based on logistic regressions; 95% confidence intervals in parentheses; p-values: * *p* < 0.05, ** *p* < 0.01, *** *p* < 0.001

### Predictors and patterns in context

#### The role of information sources, first impressions, and peer influences

Across quantitative and qualitative streams, the context of where, when and by whom initial information on the MMHW was conveyed proved exceptionally relevant. Doers mentioned that they felt compelled to enroll after hearing thoughtful and convincing explanations provided by midwives and the MMHW outreach team members, typically during ANC visits or in MMHW sensitization campaigns (see Table 2). Both midwives and outreach team members were described by doers as well as many non-doers as a trustworthy and consistent presence within participating CSBs. In the quantitative analysis, learning about the MMHW from a midwife was significantly associated with being a doer (model 3 OR = 1.667; 95%CI = 1.0588–2.6245). The statistical significance of this indicator disappeared when an indicator for learning about the MMHW from family or friends was added to the model, but the magnitude of the association remained similar (model 4 OR = 1.499; 95%CI = 0.9396–2.3927) (see Table 3). Furthermore, approval by the Malagasy Ministry of Public Health enhanced the perceived trustworthiness of the MMHW among some qualitative interview respondents.

Meanwhile, several non-doers qualitatively conveyed that they found the MMHW’s concept or premise difficult to believe or complex to understand (in terms of registration, usability and functionality), and they felt that they could not seek further clarification due to shyness and discomfort (see Table 2). MMHW outreach team members confirmed these narratives, describing how women exhibited fear in asking questions, particularly if several other women were present. While some respondents voiced that they did not understand the benefits of the MMHW and could not grasp how using the MMHW would make a difference, others explained that they arrived late for their ANC appointment or that they were told about the MMHW in a cursory fashion (while standing at a roadside) and felt they lacked an opportunity to gradually gather comprehensive information. Still other women also shared that they were uninterested in the MMHW from the start, and, therefore, did not listen attentively: “My life seemed to be just going well, and I did not enroll” (non-doer, aged 22, 2 children).

While many doers and non-doers were approached during the first months of their pregnancy, notably, non-doers mentioned that this happened only a couple of months or weeks before their baby’s due date. Some non-doers described declining enrollment because they believed they could not benefit within such a short timeframe (see Table 2).

Within communities, the rollout and implementation of the MMHW sparked lively conversations, and doers and non-doers qualitatively described how friends, neighbors, and other community members’ impressions and ideas could strongly persuade or dissuade enrollment. Respondents described routinely sharing their own choices and experiences related to enrollment and birth with other mothers and pregnant women (see Table 2). Doers, in particular, explained that their decisions to engage with the program were rooted in others’ positive previous experiences with the MMHW:

*What made me trust the MMHW was seeing my sister and our previous landlord using it*,* it served them very well […]* (doer, aged 27, 3 children)

Despite the inclusion of community health workers in the initial outreach strategy, few respondents in either data stream described learning about the MMHW from a community health worker (7.1% of respondents in the quantitative sample).

Doers and non-doers alike described rumors and concerns circulating throughout communities about the MMHW, namely that the people involved in the program could “take their money and block the SIM card afterwards” (doer, aged 21, 1 child). Qualitatively, some doers also described enrolling despite being advised against it by peers: ´

*“[The MMHW] didn’t impress me*,* because I heard [the platform] would steal […]. Later*,* when I was pregnant*,* I took the risk and thought we would see*,* because just listening to what someone is saying is not good!”* (doer, aged 22, 1 child)

Additionally, outreach team members described rumors in rural areas involving babies being stolen after MMHW usage, but such rumors were not described by mothers.

#### The role of health considerations and financial concerns

Doers and non-doers described how they wanted a birth that was ‘organized’ (doer, aged 22, 1 child), including in the event of complications or other problems that might have health-related and/or financial implications. One doer described how knowing that there was money on her MMHW brought her “mba ilaminan‘ny sainy izany” or “peace of mind” (doer, aged 21, 1 child) and eliminated fear of a frantic search for funds or assuming catastrophic debt in a worst-case-scenario.

Doers also emphasized how the MMHW facilitated a sense that they had an additional tool at their disposal to support the monitoring of their own health and that of their baby. In this regard, they particularly emphasized the MMHW’s inclusion of free ultrasound scans:

*Ultrasounds […] make a difference. In the past you waited for the birth and that is all. With the MMHW you are able to say: ‘This is my baby; it is going to be born safely’* (doer, aged 38, 4 children)

Quantitatively, having a pre-existing maternal medical risk factor such as high or low age, multiparity or complications in previous pregnancies (model 4 OR = 1.531; 95%CI = 1.0269–2.2833) was associated with being a doer. On the other hand, quantitatively, being diagnosed with maternal medical warning signs (e.g., edema, glucosuria, vaginal bleeding) within the current pregnancy was associated with being a non-doer in models 1 and 3 (model 1 OR = 0.436; 95%CI = 0.1975–0.9614; model 3 OR = 0.442; 95%CI = 0.1969–0.9931; model 4 OR = 0.454; 95%CI = 0.1981–1.0399) (see Table 3); qualitative data could not explain or elaborate a rationale for this. However, only 6.3% of women in our quantitative sample reported such medical warning signs.

Qualitatively, doers and non-doers stressed financial difficulties and described fears of engaging with a program that required a measure of trust in financial savings schemes. Respondents expressed concerns about potentially fraudulent activities when depositing money into the wallet and feeling uncertain or skeptical about whether reimbursements would be paid. Some described a fear that they might lose their SIM card where money had been saved. Furthermore, mobile money, in general, had drawbacks, including its limited accessibility in rural areas, a sense that it could facilitate theft among unscrupulous mobile money agents, and a fear that a small mistake (sending money to the wrong account) could create irreparable damage (an inability to get money back thus further burdening a family).

Quantitatively, having a higher salary (model 4 OR = 0.884; 95%CI = 0.7875–0.9914) was associated with being a non-doer (see Table 3). On the other hand, several non-doers reported in the qualitative interviews that they did not enroll because they had no money that they could initially save in the MMHW (see Table 2); other non-doers said they expected hidden registration or user fees. Doers, non-doers, and family members described how the act of placing money into a mobile platform mitigated a temptation to spend it. Outreach team members explained that some women expressed interest in enrolling before they conceived, with the intention of already saving money.

*If it is money that you don’t want to spend frivolously*,* you should put it in the phone. If you keep it at home*,* you are inevitably going to use it. Personally*,* if someone gives me money and I do not really need it*,* I put it in my phone.* (mother of a doer, aged 42)

#### The role of intra-household decision-making

Both doers and non-doers qualitatively shared varying decision-making processes concerning the MMHW; while some had directly enrolled during their ANC appointment (sometimes accompanied by a family member), others returned home and discussed with their husbands or other household members (especially their own mothers). Outreach team members described how during routine household visits, family members seemed generally supportive upon being informed about the MMHW. Conversely, in some instances, family members expressed disapproval, e.g., one husband harbored doubts about the credibility of an information source (a midwife who approached his wife on the street) (see Table 2).

When asked about decision-making habits in their household concerning healthcare seeking in general, most doers and non-doers stated that wife-husband pairs jointly make decisions. Several doers and non-doers depicted women as responsible for the health of the family and as primary decisionmakers related to healthcare seeking:

*Interviewer: “When there is a sick person at home*,* how does the discussion start*,* who is *

*involved?”*

*Respondent: “Me.”*

*Interviewer: “You? And you talk with your husband? Do you talk together?”*

*Respondent: “We discuss but I make the decision.”* (non-doer, aged 26, 1 child)

Yet in other cases (not specifically among doers or non-doers), husbands were described as primary decisionmakers on healthcare seeking (see Table 2), sometimes parents or in-laws were involved. Again, many respondents highlighted the financial implications of healthcare-related decisions. Quantitative data revealed no statistically significant differences in women’s engagement in decisions about household finances comparing doers and non-doers.

#### The role of implementation-related obstacles and opportunities

Qualitatively reported reasons for declining enrollment included plans to give birth in places where the MMHW was unavailable, such as in other regions of the country, at private facilities or with traditional birth attendants and/or at home. Some non-doers also mentioned the complexity of the enrollment process (see Table 2) or detailed how their participation in a different health-financing program deterred them from enrolling.

Despite a stated desire to enroll in the MMHW, some non-doers described facing insurmountable implementation barriers. MMHW enrollment required a national ID card, which some respondents did not bring to their ANC visits, particularly in rural areas. Several women described how they did not possess such a card. Other respondents lacked resources in terms of time and money to return to health facilities for enrollment after not taking their ID card to their initial ANC visit. A traditional *fady* (taboo) forbade one respondent from being photographed while pregnant (the MMHW-SIM-card registration required a photo). Another respondent described waiting for a planned household visit by an MMHW agent who never arrived (see Fig. 2).

Quantitative data showed no association between phone ownership and enrollment. Qualitatively, some respondents mentioned that familiarity (or a lack thereof) with mobile technology and mobile money could facilitate (or inhibit) MMHW engagement, but others argued that owning a phone was not crucial, as it was possible to save on the MMHW even without owning a phone (see Table 2).

Age restrictions related to the MMHW impeded enrollment by mothers under age 18, but outreach team members described how pregnant women who were too young to meet this criterion would occasionally use their mother’s ID card or choose a peer to enroll on their behalf to take advantage of the anticipated benefits from the MMHW.

When probed about potential improvements in outreach and sensitization, respondents explained that it would have been advantageous if they had been aware of the MMHW from the outset or even before their pregnancy. Some suggested increasing advertisements (through posters, TV, radio, SMS, and Facebook) and engaging with other family and community members. Several respondents proposed establishing a dedicated hotline for clarification purposes (they were unaware that a hotline existed). Outreach team members further suggested that allowing more than one method of identity verification could enhance accessibility to the MMHW.

## Discussion

### Main findings

This article describes factors and decision-making processes concerning enrollment for an MMHW in the Analamanga region of Madagascar. Respondents emphasized the value of receiving comprehensive information on the MMHW from a trusted source in the early stages of their pregnancy, describing it as crucial for making informed decisions. Our findings across quantitative and qualitative data streams indicate an association between learning about the MMHW from a midwife and enrolling in the program. Additionally, in many cases, peers’ prior experiences with the MMHW and family members’ viewpoints further influenced enrollment decisions. Non-doers reported barriers such as feeling less informed about the wallet, having difficulties navigating the enrollment process, or having plans to give birth elsewhere.

Most respondents (doers and non-doers) stressed the importance of health monitoring for both mother and child during pregnancy. In this respect, ultrasounds – while a valuable and important means to monitor pregnancy [51] – were described as appreciated by respondents, but the value of ultrasounds may merit enhanced sensitization as they are not a fail-safe measure (as described by at least one respondent). The association between being diagnosed with maternal medical warning signs during pregnancy and non-enrollment seemed counterintuitive to us and might result from the low proportion of women reporting such warning signs.

### Comparison to the literature

Systematic and scoping reviews have identified several factors affecting the implementation of digital health tools [52–54]: While the value of a strong patient-healthcare provider relationship and personal advice for digital health has been underscored [52, 53], insufficient advice, mistrust towards security features, and concerns about privacy were identified as barriers [54]. Implementation-related obstacles for digital health include a failure to integrate projects into the healthcare system, barriers to accessing services, a lack of knowledge and training for providers and users, low phone ownership, and network or software problems [52]. At least two of these challenges echo our findings, namely a lack of training and knowledge among users, and a sense of mistrust or incredulity about the intervention.

Our quantitative data further indicate that individuals with a higher monthly salary were less likely to enroll in the MMHW. Qualitatively, both doers and non-doers highlighted the financial costs of maternal healthcare and the medical risks of pregnancy. Overarching financial challenges were also reported in studies on maternal health wallet implementation in Zimbabwe, Kenya, Rwanda, and Madagascar, where respondents described challenges associated with low or unstable incomes, especially in the informal sector, and competing demands on limited resources (e.g., school fees, food, healthcare) [22, 35, 55]. While an unequal distribution of household income disadvantaging women, was described in Zimbabwe [55], this case was not observed in our study.

The relevance of household decision-making power and bargaining processes has been scientifically acknowledged in systematic reviews and meta-analyses across many countries [56, 57]: these processes often disadvantage women, potentially leading to adverse health outcomes [58]. However, decision-making processes vary between actors, countries, regions, and households [59]. In Antananarivo, 82.8% of women reported that they were involved in decisions on major household purchases, like national-level trends in Madagascar [6]. Furthermore, most couples in Madagascar tend to make health-related decisions jointly (woman’s health: 54.6% jointly vs. 32.1% mainly woman, 12.9% mainly husband; man’s health: 47.9% jointly vs. 34.1% mainly man, 17.3% mainly wife) [6]. Qualitative research in Madagascar that incorporated household decision-making components has presented mixed findings: In southeast Madagascar, women were often depicted as a primary authority in healthcare decisions [60]. In the Southern Highlands, the influence of husbands and other family members on the choice of birth site has been underscored [40]. In Central and Eastern regions, a variation in decision-making power concerning household finances and healthcare seeking was described [61]. These insights underscore the complexity and diversity of decision-making dynamics in different contexts and highlight a need for tailored approaches in health intervention programs.

As reported through our in-depth interviews, the variation in decision-making processes regarding healthcare seeking and MMHW adoption may explain the lack of a quantitative association between MMHW enrollment and women’s involvement in household financial decision-making. Respondents often associated health-related decisions with financial considerations. We support recommendations that maternal health projects should involve other family members [24, 40]. However, we also recognize that increasing women’s participation in household decision-making is crucial – a systematic review of qualitative studies suggests that mHealth interventions could potentially leverage this involvement [62].

Social influence has been identified as a predictor of the adoption of various digital finance and digital health tools, such as mobile wallets for general purposes in Cameroon and India [63, 64], financial technology (mobile money, mobile and internet banking) uptake in several settings in Sub-Saharan Africa [65, 66], and digital health interventions in Europe and North America [54]. The significance of an individual’s social network experiences was also observed in relation to decisions about utilizing intrauterine contraceptive devices in Madagascar [67]. Our quantitative data indicate that learning about the MMHW through familial or social interactions was associated with non-enrollment. This association may be attributed to shared negative experiences and rumors about the MMHW. However, several respondents reported that peers had motivated them to enroll, often in addition to healthcare providers. The interplay of different sources of information and influences points to the complex discourses that can arise around health (financing) programs and shape individual decisions depending on which information sources are perceived as reliable. Potential strategies to handle rumors have been discussed in a previous article on the MMHW [24].

### Limitations

Our study has limitations. First, Antananarivo constitutes the political and economic center of Madagascar; most inhabitants share higher income and better access to services [68]. The study site can therefore not be seen as representative as women in remote areas likely face different challenges [69]. We invite researchers to investigate how digital technologies can advance healthcare access in more rural areas. Second, there is a potential for recall bias since respondents could enroll in the MMHW between 2019 and 2022, while the interviews were completed in 2022. Third, this trial was conducted during the COVID-19 pandemic, which had a detrimental impact on Madagascar’s healthcare system and other sectors [70–72]. Respondents reported that the pandemic changed their everyday lives, potentially influencing their utilization of the MMHW and health services in general. Fourth, while we adjusted data collection procedures and scheduling and made efforts to mitigate selection bias, respondents who worked outside their homes may not have been as equally represented as those who worked from home or were not employed. Fifth, for qualitative sampling, we relied on phone numbers to contact respondents and, in cases where respondents did not have a personal phone, we reached out via neighbors or relatives; an approach that may have excluded those who were less digitally connected or lacked access to a community phone, also potentially biasing our sample. Finally, several relevant concepts were identified inductively during qualitative data collection and analysis. Yet, we did not design the quantitative survey to investigate these concepts fully, limiting our models’ ability to explain variation.

### Suggestions for future research

We urge future research that explores and trials alternative health financing models to improve access to care, particularly among most vulnerable populations. We also encourage implementation researchers to examine effective communication strategies that build trust and promote transparency related to the introduction and early roll-out of health interventions. In line with prior work [73], we highlight the importance of understanding social network effects on healthcare-related decisions and urge studies that explore how to harness these dynamics to improve the uptake of mobile (maternal) health financing initiatives. Related to care-seeking for maternal health and childbirth, we note that themes around risk perception and anticipated needs emerged in at least one interview and could serve as a valuable line of inquiry within future work. Finally, we underscore a need to capture the perspectives of individuals who do not enroll in public health programs despite targeted outreach and sensitization efforts.

## Conclusions

With technology permeating people’s lives globally, mobile health (financing) applications are increasingly implemented and studied. Our findings emphasize the need for sensitization activities that are thoughtfully designed and reach potential users at the right time and place. We highlight the key role of healthcare providers, family members, and one’s social network in building trust in (maternal) health financing projects. Furthermore, this study identified several implementation-related barriers, which underscores the importance of designing interventions to be as accessible as possible and to attentively embed interventions within their respective context.

## Supplementary Information

Below is the link to the electronic supplementary material.

Supplementary Material 1

Supplementary Material 2

Supplementary Material 3

Supplementary Material 4

Supplementary Material 5

## Data Availability

The datasets used and analyzed during the current study are available from the corresponding author upon reasonable request.

## Abbreviations

4MOTHERS trial: Madagascar Mobile Money for Maternal Healthcare-Related Spending trial
ANC: Antenatal care
CI: Confidence interval
CSB: Centre de santé de base
mHealth: Mobile health (use of mobile devices for healthcare delivery)
MMHW: Mobile Maternal Health Wallet
NGO: Non-governmental organization
OR: Odds ratio
