# Supplementary material for: What compels enrollment in a mobile maternal health wallet? A mixed-methods doer/non-doer analysis in Analamanga, Madagascar
Source: BMC Health Serv Res. 2025 Dec 6;25:1584. doi: 10.1186/s12913-025-13770-x (PMC12687524; doi:10.1186/s12913-025-13770-x)
Supplement: Supplementary file 1 — Supplementary Material 1 [file 12913_2025_13770_MOESM1_ESM.pdf]

Supplementary file 1: Reporting guidelines

STROBE statement (quantitative data): p.2

COREQ statement (qualitative data): p.5

STROBE Statement—Checklist of items that should be included in reports of *case-control studies*

	Item No	Recommendation	Section No
**Title and abstract**	1	(*a*) Indicate the study's design with a commonly used term in the title or the abstract	Abstract
		(*b*) Provide in the abstract an informative and balanced summary of what was done and what was found	Abstract
**Introduction**			
Background/rationale	2	Explain the scientific background and rationale for the investigation being reported	1.
Objectives	3	State specific objectives, including any prespecified hypotheses	1.
**Methods**			
Study design	4	Present key elements of study design early in the paper	2.3.
Setting	5	Describe the setting, locations, and relevant dates, including periods of recruitment, exposure, follow-up, and data collection	2.1., 2.2., 2.4.1
Participants	6	(*a*) Give the eligibility criteria, and the sources and methods of case ascertainment and control selection. Give the rationale for the choice of cases and controls	2.4.1
		(*b*) For matched studies, give matching criteria and the number of controls per case	not applicable
Variables	7	Clearly define all outcomes, exposures, predictors, potential confounders, and effect modifiers. Give diagnostic criteria, if applicable	2.4.2.
Data sources/ measurement	8	For each variable of interest, give sources of data and details of methods of assessment (measurement). Describe comparability of assessment methods if there is more than one group	2.4.1.

Bias	9	Describe any efforts to address potential sources of bias	4.3.
Study size	10	Explain how the study size was arrived at	2.4.2., supplementary file 3
Quantitative variables	11	Explain how quantitative variables were handled in the analyses. If applicable, describe which groupings were chosen and why	2.4.2.
Statistical methods	12	(*a*) Describe all statistical methods, including those used to control for confounding	2.4.2.
		(*b*) Describe any methods used to examine subgroups and interactions	not applicable
		(*c*) Explain how missing data were addressed	2.4.2
		(*d*) If applicable, explain how matching of cases and controls was addressed	not applicable
		(*e*) Describe any sensitivity analyses	not applicable
**Results**			
Participants	13*	(a) Report numbers of individuals at each stage of study—eg numbers potentially eligible, examined for eligibility, confirmed eligible, included in the study, completing follow-up, and analysed	2.4.2
		(b) Give reasons for non-participation at each stage	2.4.2
		(c) Consider use of a flow diagram	not applicable
Descriptive data	14	(a) Give characteristics of study participants (eg demographic, clinical, social) and information on exposures and potential confounders	3.1.
		(b) Indicate number of participants with missing data for each variable of interest	supplementary file 3
Outcome data	15*	Report numbers in each exposure category, or summary measures of exposure	3.1.
Main results	16	(*a*) Give unadjusted estimates and, if applicable, confounder-adjusted estimates and their precision	3.3., 3.4.

		(eg, 95% confidence interval). Make clear which confounders were adjusted for and why they were included	
		(*b*) Report category boundaries when continuous variables were categorized	3.3.
		(*c*) If relevant, consider translating estimates of relative risk into absolute risk for a meaningful time period	not applicable
Other analyses	17	Report other analyses done—eg analyses of subgroups and interactions, and sensitivity analyses	3.3.
**Discussion**			
Key results	18	Summarise key results with reference to study objectives	4.1.
Limitations	19	Discuss limitations of the study, taking into account sources of potential bias or imprecision. Discuss both direction and magnitude of any potential bias	4.3.
Interpretation	20	Give a cautious overall interpretation of results considering objectives, limitations, multiplicity of analyses, results from similar studies, and other relevant evidence	4.1.-4.3.
Generalisability	21	Discuss the generalisability (external validity) of the study results	not applicable
**Other information**			
Funding	22	Give the source of funding and the role of the funders for the present study and, if applicable, for the original study on which the present article is based	2.4.1., Declarations

*Give information separately for cases and controls.

Based on: Elm E von, Altman DG, Egger M, Pocock SJ, Gøtzsche PC, Vandenbroucke JP. The Strengthening the Reporting of Observational Studies in Epidemiology (STROBE) statement: guidelines for reporting observational studies. PLoS Med 2007; 4(10):e296.

COREQ: Consolidated criteria for reporting qualitative research: a 32-item checklist for interviews and focus groups

Section/Topic	Item No	Checklist item	Section No / additional information
**Domain 1: Research team and reflexivity**			
Personal Characteristics			
Interviewer/facilitator	1	Which author/s conducted the interview or focus group? Interviewer/facilitator	MLR conducted all interviews except one interview with one implementation team member (conducted by VSC, see acknowledgements)
Credentials	2	What were the researcher's credentials? E.g. PhD, MD	Bachelor's degree (or equivalent): LNS, MLR, LMT; master's degree: ER, JVE; MD: ZR, SK, JVE, HEA, BV, TB; PhD: LB, TB, MDCR, SAM
Occupation	3	What was their occupation at the time of the study?	MLR was employed by the implementation partner *Doctors for Madagascar*, all other research team members were employed by or/and doctoral/master's students at their respective institutions
Gender	4	Was the researcher male or female?	The research team included seven female, five male and one non-binary individual. In-depth interviews were conducted by female team members.
Experience and training	5	What experience or training did the researcher have? Relationship with participants	2.5.2.
Relationship with participants			
Relationship established	6	Was a relationship established prior to study commencement?	2.5.2.
Participant knowledge of the interviewer	7	What did the participants know about the researcher? e.g. personal goals, reasons for doing the research	2.5.2. , respondents were informed prior to the interview that research was conducted to improve the MMHW and to understand why some people

			decided to use the intervention while others did not.
Interviewer characteristics	8	What characteristics were reported about the interviewer/facilitator? e.g. Bias, assumptions, reasons and interests in the research topic	The interviewers had no affiliation with the MMHW project prior to the conduction of the qualitative study.
**Domain 2: study design**			
Theoretical framework			
Methodological orientation and Theory	9	What methodological orientation was stated to underpin the study? e.g. grounded theory, discourse analysis, ethnography, phenomenology, content analysis	2.5.3.
Participant selection			
Sampling	10	How were participants selected? e.g. purposive, convenience, consecutive, snowball	2.5.1.
Method of approach	11	How were participants approached? e.g. face-to-face, telephone, mail, email	2.5.2.
Sample size	12	How many participants were in the study?	figure 1, 3.1.
Non-participation	13	How many people refused to participate or dropped out? Reasons?	2.5.1.
Setting of data collection	14	Where was the data collected? e.g. home, clinic, workplace	2.5.2.
Presence of non-participants	15	Was anyone else present besides the participants and researchers?	2.5.2.
Description of sample	16	What are the important characteristics of the sample? e.g. demographic data, date	3.1.
Data collection			
Interview guide	17	Were questions, prompts, guides provided by the authors? Was it pilot tested?	2.5.2.
Repeat interviews	18	Were repeat interviews carried out? If yes, how many?	2.5.1., figure 1
Audio/visual recording	19	Did the research use audio or visual recording to collect the data?	2.5.2.
Field notes	20	Were field notes made during and/or after the interview or focus group?	2.5.3.
Duration	21	What was the duration of the interviews or focus group?	2.5.2.
Data saturation	22	Was data saturation discussed?	2.5.2.

Transcripts returned	23	Were transcripts returned to participants for comment and/or correction?	Logistical and financial constraints inhibited returning transcripts to participants
**Domain 3: analysis and findings**			
Data analysis			
Number of data coders	24	How many data coders coded the data?	2.5.3.
Description of the coding tree	25	Did authors provide a description of the coding tree?	The development process of the codebook is described in 2.5.3. The inductively developed codebook contained codes about: Initial impressions about the MMHW, personal considerations and household decision-making, peer's perspectives, general attitudes towards technology and healthcare seeking, saving habits, trust into the MMHW utilization of the MMHW and potentials for improvement (doers).
Derivation of themes	26	Were themes identified in advance or derived from the data?	2.5.3.
Software	27	What software, if applicable, was used to manage the data?	2.5.3
Participant checking	28	Did participants provide feedback on the findings?	Logistical and financial constraints inhibited participant checking
Reporting			
Quotations presented	29	Were participant quotations presented to illustrate the themes / findings? Was each quotation identified? e.g. participant number	3.4.1.-3.4.4., table 3 / identifiers for each quote can be found in supplementary file 4
Data and findings consistent	30	Was there consistency between the data presented and the findings?	3.2.-3.4.4., 4.1.-4.2.
Clarity of major themes	31	Were major themes clearly presented in the findings?	3.2.-3.4.4., 4.1.-4.2.
Clarity of minor themes	32	Is there a description of diverse cases or discussion of minor themes?	3.2.-3.4.4., 4.1.-4.2.

Based on: Tong A, Sainsbury P, Craig J. Consolidated criteria for reporting qualitative research (COREQ): a 32-item checklist for interviews and focus groups. Int J Qual Health Care 2007; 19(6):349–57.
