# Supplementary material for: What compels enrollment in a mobile maternal health wallet? A mixed-methods doer/non-doer analysis in Analamanga, Madagascar
Source: BMC Health Serv Res. 2025 Dec 6;25:1584. doi: 10.1186/s12913-025-13770-x (PMC12687524; doi:10.1186/s12913-025-13770-x)
Supplement: Supplementary file 2 — Supplementary Material 2 [file 12913_2025_13770_MOESM2_ESM.pdf]

Supplementary file 2: In-depth interview guides

Cover sheets for all interviews (English) p.2

Cover sheets for all interviews (Malagasy) p.6

Doers/non-doers (English) p.10

Doers/non-doers (Malagasy) p.17

Household members (English) p.26

(Note: As the interview guide for household team members only included minor changes compared to those for doers/non-doers, changes to the Malagasy version were discussed orally by the research team before each interview).

Implementation team members (English/Malagasy) p.32

Cover sheet and Interview guide
4MOTHERS Doer/Non-doer analysis
version August 11th, 2022 – 3.1

Main research question:
Who registers for a Maternal Health Wallet based on mobile money and who doesn't?

Cover sheet (4 pages)

Interview Identification

I1	Interview Number	_______
I2	Name of Interviewer	___________________________________
I3	Interview Date	___ . ___ . ______ (dd.mm.yyyy)
I4	Time Start	___:___
**I5**	**Time End**	**___:___**
**I6**	**Outcome of Interview**	**☐ Complete**<br>**☐ Incomplete, reason:**<br>**_______________________________**
**I7**	**Was the interview interrupted? (another person coming in, noises etc.)**	**☐ No**<br>**☐ Yes - by whom, how and why?**<br>**_______________________________**<br>**_______________________________**

bold: to be filled out after the interview

Respondent profile

R1	Interview partner	☐ Mother<br>☐ Other household member: ____________________<br>☐ Other person: ______________________________
R2	User / Non-User	☐ User<br>☐ non-User<br>☐ Does not apply: ____________________________
R3	Relationship status:	☐ Single<br>☐ Partner, living together<br>☐ Married<br>☐ Separated<br>☐ Other: __________________
R4	Area	☐ Rural<br>☐ Urban<br>☐ Other: ___________________
R5	Risk factors / Complications (tick all that apply)	☐ None<br>☐ Risk factor(s) (see list): _______________________<br>☐ Complication(s) (see list): _____________________
R6	Age	____ years old
**R7**	**Occupation**	_________________________

bold: to be filled out after the interview

**Interviewer comments:**
What are your impressions of the interview? Did you observe something special? Was there anything surprising / new for you?

Were there any problems with the interview guide or the related documents?<br>Was the place for the meeting point chosen well? Is there anything else we could consider changing?
Did the participant mention any contacts for snowball sampling?

**Reflection**

How do you think this interview went? How difficult was this interview for you?
How much do you feel that you affected this interview? What else would be important to add?

Pejy fonony sy torolalana amin'ny dinidinika

“4Mothers” Famakafakana momba ny Mpanatanteraka/tsy mpanantanteraka

Fanovana ny 11 Aogositra 2022 – 3.0

Fanontaniana fikarohana lehibe:

Iza no misoratra anarana amin'ny Tahiry momba ny fahasalaman'ny reny mifototra amin'ny finday ary iza no tsy manao izany?

Taratasy fanoratana (pejy 4)

Fanadihadiana famantarana

I1	Laharan’ny fanadihadiana	_______
I2	Anaran'ny mpanadihady	___________________________________
I3	Datin’ny dinidinika	___ . ___ . ______ (dd.mm.yyyy)
I4	Ora fanombohana	___:___
**I5**	**Ora famaranana**	**___:___**
**I6**	**Ny vokatry ny dinidinika**	☐ **feno**<br>☐ **Tsy feno, antony:**<br>______________________________
**I7**	**Tapaka ve ny tafatafa? (olona hafa miditra, mitabataba sns.)**	☐ **tsy misy**<br>☐ **Eny - avy amin'iza, amin'ny fomba ahoana ary nahoana?**<br>______________________________<br>______________________________

Soratra vaventy: fenoina aorian'ny dinidinika

<u>Mombamomba ny mpamaly</u>

R1	Miara-midinika	☐ Reny<br><br>☐ Olona hafa ao an-tokantrano: ______________________<br><br>☐ Olona hafa: _________________________________
R2	Mpampiasa / tsy mpampiasa	☐ mpampiasa<br><br>☐ tsy mpampiasa<br><br>☐ Tsy mihatra: _______________________________
R3	Toe-panambadiana:	☐ Mpitovo<br><br>☐ Fofom-bady, miara-monina<br><br>☐ Manambady<br><br>☐ Misaraka<br><br>☐ Hafa: _______________________
R4	Faritra	☐ Ambanivohitra<br><br>☐ Tanàna<br><br>☐ Hafa: _____________________
R5	Antony mety hampidi-doza /<br><br>Fahasarotana<br><br>(mariho izay mihatra rehetra)	☐ tsy misy<br><br>☐ Antony mety hampidi-doza (jereo ny lisitra): _________________________<br><br>☐ Fahasarotana (jereo ny lisitra): _____________________
R6	Taona	_____ taona
**R7**	**Asa**	___________________________

**Fanehoan-kevitry ny mpanadihady:**
Inona no tsapanao momba ny tafatafa? Nahita zavatra manokana ve ianao? Nisy zavatra mahagaga/vaovao ho anao ve?

Nisy olana ve tamin'ny torolalana fanadihadiana na ireo antontan-taratasy mifandraika amin'izany?<br><br>Voafidy tsara ve ny toerana nanaovana ilay fivoriana? Misy zavatra hafa tokony eritreretina hovaina ve?
Moa ve ny mpandray anjara nilaza ny fifandraisany tamin'ny fanadihadiana teny anivon'ny fiaraha-monina?

**Traika**

Nanao ahoana araka ny hevitrao ny fizotran'ity tafatafa ity? Nanao ahoana ny fahasarotan'ity tafatafa ity ho anao?

Inona no tsapanao fa nisy fiantraikany tamin'ity tafatafa ity? Inona koa no zava-dehibe tokony hanampiana izany?

Semi-structured Interview guide: doers/non-doers

Thank you very much for agreeing to participate in another interview for our study on mTOMADY and the Mobile Maternal Health Wallet. Our aim in this part of the study is to understand why some people decide to use the intervention while others do not and especially how these decisions are made. Our goal is to improve the intervention to make it more accessible and beneficial.

I would now like to address a few general things about the project and the interview itself before we really get into it: First, as we described in the information sheet, participation is anonymous. This means that we make sure that all statements and information, that could enable somebody to identify you, are not linked to the answers you give in the interview. Participation is entirely voluntary. You can refuse to answer specific questions that you do not want to answer and you can stop the interview at any time. Please also let us know if you do not want to answer a specific question. To be able to properly analyse the interview later, we also need to record it. You can interrupt, restart or cancel the recording at any time. Therefore, you can use the buttons on the tablet. Do you have any further questions about it? Can I start the recording?

[START RECORDING]

Thank you! I would now like to talk with you about your experiences, perceptions and views on the Mobile Maternal Health Wallet including mTOMADY. There are no right and wrong answers, it is all about your personal experiences, views, and perceptions. We ask you to tell us everything you can think of.

**TOPIC 1:** "Ice-breaking question" and first thoughts<br><br>*This section should help to start a conversation, especially about the decision-making process.*	
1.1	First of all: How is your baby doing?
1.2	Please try to remember the first time somebody told you about mTOMADY. Explain the situation to me so I can feel a bit how you felt.<br><br>*Probes:*<br>Where were you?<br>When was it?<br>Who told you about it?<br>What were the first feelings and thoughts about it?<br>How did it go on afterwards?

Reminder: Ask probes like „Can you tell me more about this“? / "Then what happened?"

**TOPIC 2:** *Decision-making-process: personal perception, influences of household members and other people*<br><br>*The first questions focus on the personal perception of the decision-making process and main reasons, perceived advantages and disadvantages. Further, some questions aim to understand more about the women's environment and who influenced the decision.*	
2.1	*Doers:* Please try to remember how you thought about it and finally decided to register for mTOMADY. Please tell me the story of how the decision to register was taken.<br><br>*Non-doers:* We know that many people didn't sign up for mTomady. We can't talk to all of them but I can talk to you. Therefore, we would be happy if you could share some insights why you / your household didn't use the intervention: Please try to remember how you thought about it and finally decided not to register for mTOMADY. Please tell me the story of how the decision not to register was taken.
2.2	Who took the final decision?<br><br>*Probes:*<br>Please describe when the decision to use mTOMADY was taken during your pregnancy?<br>Why did your household decide at this point of time?
2.3	Which reasons would you consider as the main ones for the decision?
2.4	What were the advantages for you?
2.5	What were the disadvantages for you?
2.6	Please explain how far your decision was influenced by members of your household if anybody had an influence.<br><br>*Probes:*<br>What was this person's opinion?<br>Why did this person's opinion influence your decision?<br>How large was this person's influence?<br>What would be the consequence if you would have decided against this person's opinion?

	Who else in your household had an influence on the decision?<br>Who tried to convince you to use mTOMADY?<br>Was there anybody who told you not to use mTOMADY?
2.7	Please explain how far your decision was influenced by other people who are not members of your household.<br><br>*Probes:*<br>What was this person's opinion?<br>Why did this person's opinion influence your decision?<br>How large was this person's influence?<br>Who else had an influence on the decision?<br>Who tried to convince you to use mTOMADY?<br>Was there anybody who told you not to use mTOMADY?
2.8	We already heard that in some cases, there are community health workers that recommend mTOMADY and some not so much. How has the relation to a community health worker influenced your decision (not) to register?<br><br>*Probe:*<br>How is your relationship with your community health worker?<br>How good do you know the community health worker in your community?<br>How has the community health worker influenced your decision?<br>In which ways has the community health worker recommended mTOMADY or not?
2.9	If you could decide again now, how would you decide? And why?

Reminder: Silence is a good probe

**TOPIC 3:** *Healthcare and healthcare expenses*<br><br>*These questions aim to help us to understand how the women we are interviewing are used to seeking healthcare, how they save for these purposes and if using mTOMADY changed something about this.*	
3.1	I would now like to talk to you about healthcare in general. Please think of a situation when you need to seek healthcare. Please explain the process that happens until you seek healthcare.<br><br>*Probes:*<br>How does such a discussion start?<br>Who is generally involved in deciding whether you will seek healthcare?<br>Where would you usually seek care? Why would you seek care there?

	If there are any, please tell me about other places where you would seek care.<br>Please describe factors that would keep you from seeking care when you need it.
3.2	How would you describe the service you receive for healthcare?<br><br>*Probes:*<br>How do you feel that your needs are met?<br>What do you think of the time that is spent with you?<br>How do you feel about the attention that is given to you?<br>What do you think of the medication they give to you?
3.3	How do you usually save for healthcare purposes if you do so?<br><br>*Probes:*<br>Which tools do you use for savings?<br>How often do you save?<br>For which situations do you save?
3.4	How have you been saving for your pregnancy if you saved at all? Why did you do it like this?
3.5	*Doers only:* Getting back to mTOMADY: How did the way you save change by using mTOMADY if it changed?<br><br>*Probe:*<br>If it was not your first pregnancy, what made saving different from pregnancies before if it did at all?

Reminder: Probes can also be phrased like: "If you would compare x and y, what differences would you see?"

**TOPIC 4:** mTOMADY Barriers and improvements / Mobile Money<br><br>*These questions aim less on the decision-making but on general perception about the intervention, areas for improvement & trust in mTOMADY.*	
4.1	*Doers:* mTOMADY was meant to help women to receive better care during pregnancy and childbirth. We know it's more helpful in some cases and in some less. How about you?

	Probes Doers:<br>If you had children before: How was it different to have used mTOMADY compared to your pregnancies before if it was different at all?<br>How did you use mTOMADY?<br><br>*Non-doers:* mTOMADY was meant to help women to receive better care during pregnancy and childbirth. We know it's more helpful in some cases and in some less. If you had used mTomady, how do you think it would have been for you? Why? / why not?
4.2	How would you describe your feelings about mTOMADY over the time of your pregnancy?<br><br>*Probes:*<br>Please explain if your feelings about mTomady changed.<br>What made these feelings change?<br>What was different between the beginning and the end of your pregnancy?
4.3	If you would describe mTomady to another person, what would you tell them?<br><br>Probes:<br><br>If you would describe how you can check how many money you have on your mT, how would you do that?<br>Please explain where you you get other information about mT from?
4.4	What information did you wish to have had earlier?<br>How would you have liked to have learned about mT?<br><br>How is the easiest way for your pesonal case to get information?<br>Probes: In the health facility, community health worker, video, internet, community agents,<br><br>When would you have liked to have learned about mT?<br><br>Who should be addressed by mTomady?<br>Probes: Who from your family? Who from your community?<br><br>How can we also inform your [people mentioned].
4.5	*NOTE: Tell participant that there are 4 questions left.*<br><br>Our aim is to make mTOMADY more useful to women. What could we do better? How did we make mistakes?

	Probes:<br>What are barriers for registration in your opinion?<br>What are barriers for usage in your opinion?<br>For whom do you think mTOMADY is more and for whom less helpful?<br>What additional information you would have liked to have but didn't receive?
4.6	mTOMADY works using mobile money technology. We know that some people find it easier, some more difficult to use. What do you think about it?<br><br>*Probes:*<br>What makes it easy and what makes it difficult for you to use mobile money?<br>What are problems with using mobile money?
4.7	Please explain what makes you trust or distrust mTOMADY.
4.8	Who profits most from mTOMADY from your point of view?<br><br>*Probe:*<br>Who doesn't profit from mTOMADY?
4.9	One of the things we heard was that some people were nervous about how what happens with their money. What do you think about it?<br><br>IF YES: What makes you nervous about your money?<br><br>We have heard from other people thought that mT might be a scam? What have you thought about this?<br><br>What is your personal definition of a scam?

Reminder: Ask probes like: "What else?" / "Is there anything else?"

**TOPIC 5:** *Community perception*<br>*These questions aim to contribute to the understanding of how people in the community, who are not household members of the respondent, perceive the intervention.*	
5.1	Finally, we want to learn more about what different people think about mTOMADY. Would you please explain how people in your community talk about it?

	Probes:<br>What did this person say to you?<br>What is this person's role in your community?<br>What are the very positive opinions in your community?<br>What are the very negative opinions in your community?<br>What words do people use to describe mTOMADY?<br>What do you think about them using these words?<br>How was this discussed when you were at the health centre?<br>We already heard that in some places there were rumours about mTOMADY. If you heard of any, would you please describe them?

**TOPIC 6:** End of the interview	
6.1	Is there anything important we have not talked about yet from your perspective? Anything you would like to add?
6.2	Is there anything I didn't ask you that I should have asked you?
6.3	Do you have any questions for me?

Thank you very much for participating!

[STOP RECORDING]

<u>Snowball sampling</u>

Do you know somebody who would be interesting to interview for us?
Do you know somebody who had a special experience (positive or negative) with MMHW?
Coming back to the questions to who influenced you: Do you think we could maybe talk to [....] as well?
Coming back to the question about different opinions in your community: Do you think we could maybe talk to [....] as well?
Could we get back to you again to get in contact with this person?

“4Mothers” Famakafakana momba ny Mpanatanteraka/tsy mpanantanteraka

Fanovana ny 16 Septambra 2022 – 3.1

Torolàlana fanadihadiana voarindra

Misaotra betsaka anao nanaiky handray anjara amin'ity tafatafa iray hafa ity momba ny fandalinanay ny mTOMADY sy ny fitahirizam-bola amin’ny finday ho an’ny fahasalaman’ny Reny. Ny tanjonay amin'ity ampahany amin'ny fandalinana ity dia ny hahatakaranay ny antony nanapahan'ny olona sasany ny fampiasana izany, ary ny hafa kosa tsy manao izany, ary indrindra ny fomba fandraisana ireo fanapahan-kevitra ireo. Ny tanjonay dia ny hanatsara ny tolotra mba hahatonga azy ho mora sy mahasoa kokoa.

Te hiresaka zavatra ankapobeny vitsivitsy momba ny tetikasa sy ny dinidinika manokana aho izao, alohan'ny tena hidirantsika amin'izany: Voalohany, araka ny nofaritanay tao amin'ny takelaka fampahafantarana, ny fandraisana anjara dia tsy fantatra anarana. Midika izany fa ataonay azo antoka fa ny fanambarana sy fampahalalana rehetra, izay mety hahafahan'ny olona iray hamantatra anao, dia tsy mifandray amin'ny valiny omenao amin'ny tafatafa. An-tsitrapo tanteraka ny fandraisana anjara. Azonao atao ny mandà tsy hamaly fanontaniana manokana izay tsy tianao hovaliana ary azonao atao ny manajanona ny tafatafa amin'ny fotoana rehetra. Ampahafantaro anay koa azafady raha tsy te hamaly fanontaniana manokana ianao. Mba hahafahana mamakafaka tsara ny dinidinika any aoriana dia mila mandray feo izany koa izahay. Azonao atao ny manapaka, mamerina na manafoana ny fandraisam-peo amin'ny fotoana rehetra. Noho izany, azonao atao ny mampiasa ny bokotra eo amin'ny fandraisam-peo. Manana fanontaniana fanampiny momba izany ve ianao? Afaka manomboka ny fandraisam-peo ve aho?

[Atomboka ny fandraisam-peo]

Misaotra anao! Te hiresaka aminao momba ny traikefanao, ny fandraisanao ary ny fomba fijerinao momba ny Tahiry amin’ny finday itsinjovana ny fahasalaman’ny Reny aho, ao anatin'izany ny mTOMADY. Tsy misy valiny marina sy diso fa miankina amin'ny traikefanao manokana sy ny fomba fijerinao ary ny fiheveranao izany. Miangavy anao izahay mba hilaza aminay izay rehetra ao an-tsainao momba izany.

LOHAHEVITRA 1 : “Sava ranon’ando” sy fanombohana<br><br>*Ity fizarana ity dia tokony hanampy amin'ny fanombohana resadresaka, indrindra momba ny fizotran'ny fanapahan-kevitra.*	
1.1	Voalohany indrindra: manao ahoana ny zanakao?

1.2	Azafady, tadidio ny fotoana voalohany nisy olona nilaza taminao momba ny mTOMADY. Hazavao amiko ny zava-nisy mba hahafahako mahatsapa kely ny fihetseham-ponao.<br><br>Fanadihadiana:<br><br>Taiza ianao?<br><br>Oviana izany?<br><br>Iza no nilaza izany taminao?<br><br>Inona no fihetseham-po sy eritreritra voalohany momba izany?<br><br>Ahoana no fizotrany taorian'izay?

Fampahatsiahivana: Manontania fandalinana toy ny hoe "Afaka milaza amiko bebe kokoa momba an'io ve ianao"? / "Dia inona no nitranga?"

LOHAHEVITRA 2: Dingan'ny fandraisana fanapahan-kevitra: ny hevitry ny tena manokana, ny fitaoman'ny olona ao an-tokantrano sy ny olon-kafa<br><br>*Ny fanontaniana voalohany dia mifantoka amin'ny hevitry ny tena manokana momba ny dingan'ny fandraisana fanapahan-kevitra sy ny antony lehibe, ny tombony sy ny fatiantoka hita. Fanampin'izany, ny fanontaniana sasany dia mikendry ny hahatakarana bebe kokoa momba ny tontolo iainan'ny vehivavy sy izay nandrisika tamin'ny fanapahan-kevitra.*	
2.1	Mpanatanteraka: Mba ezaho tsiahivina ny fomba nieritreretanao momba izany ary farany ny nanapahana hevitra ny hisoratra anarana ao amin'ny mTOMADY. Mba lazao amiko ny fomba nandraisana ny fanapahan-kevitra hisoratra anarana.<br><br>Tsy mpanatanteraka: Fantatray fa betsaka ny olona tsy nisoratra anarana tamin'ny mTomady. Tsy afaka miresaka amin'izy rehetra izahay fa afaka miresaka aminao aho. Noho izany dia ho faly izahay raha afaka mizara fomba fijery vitsivitsy ianao hoe nahoana ianao / ny ankohonanao no tsy nampiasa ny tolotra: Azafady, ezaho tsiahivina ny fomba nieritreretanao momba izany ary farany ny nanapahana hevitra ny tsy hisoratra anarana ao amin'ny mTOMADY. Mba lazao amiko ny fomba nandraisana ny fanapahan-kevitra tsy hisoratra anarana.
2.2	Iza no nandray ny fanapahan-kevitra farany?<br><br>Fanadihadiana:<br><br>Lazao azafady hoe oviana no nandraisana ny fanapahan-kevitra hampiasa ny mTOMADY nandritra ny fitondranao vohoka?

	Nahoana ianao na ny tao an-tokatranonao no nandray izay fanampahankevitra izay?
2.3	Inona no antony heverinao ho lehibe indrindra tamin'ny fanapahan-kevitra?
2.4	Inona no tombony ho anao?
2.5	Inona no fatiantoka ho anao?
2.6	Azafady, hazavao hoe hatraiza ny fanapahan-kevitrao no voataon'ny mpikambana ao amin'ny ankohonanao, raha nisy fiantraikany avy tamin'olona.<br><br>Fanadihadiana:<br><br>Inona no hevitr'io olona io?<br><br>Nahoana no nisy fiantraikany tamin'ny fanapahan-kevitrao ny hevitr'io olona io?<br><br>Manahoana ny haben'ny fitaoman'io olona io?<br><br>Inona no mety ho vokany raha toa ka nanapa-kevitra hanohitra ny hevitr'io olona io ianao?<br><br>Iza koa no tao an-tokantranonao no nanan-kery tamin'ilay fanapahan-kevitra?<br><br>Iza no nanandrana nandresy lahatra anao hampiasa mTOMADY?<br><br>Sao mba nisy niteny taminao hoe aza mampiasa mTOMADY?
2.7	Azafady, hazavao hoe hatraiza ny fiantraikan'ny olona hafa tsy mpikambana ao amin'ny ankohonanao tamin'ny fanapahan-kevitrao.<br><br>Fanadihadiana:<br><br>Inona no hevitr'io olona io?<br><br>Nahoana no nisy fiantraikany tamin'ny fanapahan-kevitrao ny hevitr'io olona io?<br><br>Hatraiza ny halehiben'ny fitaoman'io olona io?<br><br>Iza koa no nanan-kery tamin'ilay fanapahan-kevitra?

	Iza no nanandrana nandresy lahatra anao hampiasa mTOMADY?<br><br>Sao mba nisy niteny taminao hoe aza mampiasa mTOMADY?
2.8	Efa renay fa amin’ny tranga sasantsasany, misy mpiasan'ny fahasalamam-bahoaka mandrisika momba ny mTOMADY, ny sasany kosa tsy dia toy izany loatra. Inona no fiantraikan'ny fifandraisana amin'ny mpiasan'ny fahasalamana eny amin’ny fiarahamonina tamin'ny fanapahan-kevitrao (tsy) hisoratra anarana?<br><br>Fandalinana:<br><br>Ahoana ny fifandraisanao amin'ny mpiasan'ny fahasalamana eo amin'ny fiaraha-monina misy anao?<br><br>Hatraiza ny fahafantaranao ny mpiasan'ny fahasalamana eo amin'ny fiaraha-monina misy anao?<br><br>Ahoana no fiantraikan'ny mpiasan'ny fahasalamana eo amin'ny fiaraha-monina tamin'ny fanapahan-kevitrao?<br><br>Amin'ny fomba ahoana no nanoroan'ny mpiasan'ny fahasalamam-bahoaka ny mTOMADY sa tsia?
2.9	Raha afaka manapa-kevitra indray ianao izao, ahoana ny anapahanao hevitra? Ary nahoana?

Fampahatsiahivana: Fandalinana tsara ny fahanginana

LOHAHEVITRA 3: Ny fitsaboana sy fandaniana ara-pitsaboana<br><br>*Ireto fanontaniana ireto dia mikendry ny hanampy antsika hahatakatra ny fomba nampiasan'ireo vehivavy hadihadianay amin'ny fikarakarana ara-pahasalamana, ny fomba fitahirizan’izy ireo ho amin’izany, ary raha nanova zavatra momba izany ny fampiasany mTOMADY.*	
3.1	Te hiresaka aminao momba ny fitsaboana amin'ny ankapobeny aho izao. Mba eritrereto ny toe-javatra iray izay ilanao fikarakarana ara-pahasalamana. Hazavao azafady ny dingana mitranga mandra-pikarohanao fitsaboana.<br><br>Fanadihadiana:<br><br>Ahoana no fiaingan’ny dinika toy izany?<br><br>Iza amin'ny ankapobeny no mandray anjara amin'ny fanapahan-kevitra raha hitady fitsaboana ianao?

	Aiza no hitadiavanao fitsaboana? Nahoana ianao no mitady fitsaboanana any?<br><br>Raha misy dia mba lazao ahy ny momba ny toerana hafa hitadiavanao fitsaboana.<br><br>Azafady, lazao ireo antony mety hisakana anao tsy hitady fitsaboana rehefa mila izany ianao.
3.2	Ahoana no hamaritanao ny tolotra azonao amin'ny fitsaboana?<br><br>Fanadihadiana:<br><br>Ahoana no fiheveranao fa voavaly ny filanao?<br><br>Ahoana ny hevitrao momba ny fotoana lany miaraka aminao?<br><br>Inona no tsapanao momba ny fiheverana anao?<br><br>Ahoana ny hevitrao momba ny fanafody omeny anao?
3.3	Ahoana no fanaonao mazàna raha mitahiry vola ho an'ny tanjona ara-pahasalamana ianao?<br><br>Fanadihadiana:<br><br>Inona no fitaovana ampiasainao amin'ny fitahirizam-bola?<br><br>Impiry ianao no mametraka tahiry?<br><br>Amin'ny toe-javatra inona no itahirizanao vola?
3.4	Nanao ahoana ny fomba fitahirizanao vola ho an'ny fitondrana vohoka raha nanangona mihitsy ianao? Nahoana ianao no nanao toy izany?
3.5	Ho an’ny mpanatanteraka ihany: Miverina amin'ny mTOMADY: Ahoana ny fiovan’ny ny fomba fitahirizanao vola tamin'ny fampiasana mTOMADY raha niova izany?<br><br>Fanadihadiana:<br><br>Raha toa tsy fitondranao vohoka voalohany izany, inona no tena nampiavaka ny fitahirizana vola mitaha tamin'ny fitondrana vohoka teo aloha?

Fampahatsiahivana: Ny fanontaniana dia azo adika koa hoe: "Raha ampitahainao ny “x” sy “y”, inona no fahasamihafana hitanao?"

LOHAHEVITRA 4: mTOMADY Sakana sy fanatsarana / Fitahirizana amin'ny finday<br><br>*Ireto fanontaniana ireto dia tsy mikendry loatra ny fandraisana fanapahan-kevitra fa amin'ny fomba fijery ankapobeny momba ny tolotra, ny lafiny fanatsarana sy ny fahatokisana ny mTOMADY.*	
4.1	Mpampiasa: Ny mTOMADY dia natao hanampiana ny vehivavy hahazo fikarakarana tsara kokoa mandritra ny fitondrana vohoka sy ny fiterahana. Fantatsika fa manampy kokoa izany amin'ny toe-javatra sasany ary amin'ny tranga sasany somary latsaka kokoa. Ahoana ny hevitrao?<br><br>Fanadihadiana ny Mpampiasa:<br><br>Raha nanan-janaka ianao taloha: Inona no mahasamihafa ny nampiasa mTOMADY raha oharina amin'ny fitondrana vohoka teo aloha raha toa ka tena samihafa mihitsy?<br><br>Ahoana no nampiasanao mTOMADY?<br><br>Tsy mpampiasa: Natao hanampiana ny vehivavy ny mTOMADY mba hahazo fikarakarana tsara kokoa mandritra ny fitondrana vohoka sy ny fiterahana. Fantatsika fa manampy kokoa izany amin'ny toe-javatra sasany ary amin'ny tranga sasany somary latsaka kokoa. Raha nampiasa mTomady ianao, araka ny hevitrao, ahoana no mety kokoa ho anao? Nahoana? / Nahoana no tsy?
4.2	Ahoana no hamaritanao ny fihetseham-ponao momba ny mTOMADY nandritra ny fotoana nitondranao vohoka?<br><br>Fanadihadiana:<br><br>Hazavao azafady raha niova ny fihetseham-ponao momba ny mTomady.<br><br>Inona no nahatonga ireo fahatsapana ireo niova?<br><br>Inona no maha samy hafa ny fiandohana sy ny fiafaran'ny fitondranao vohoka?
4.3	Te hijery miaraka aminao izahay ahoana ny hanatsarana ny fampahafantarana ny mTOMADY. Raha hiresaka momba ny mTOMADY amin'ny olana hafa ianao, inona no ho lazainao?<br><br>Afaka hazavainao kokoa ve?
4.4	Raha misy olon-kafa manontany anao mikasika ny fomba fametrahana vola ao anatin'ny kaonty mTOMADY,, afaka ho resahinao amiko ve ny ho resahinao aminy?

	Raha misy olon-kafa manontany anao mikasika ny fomba hafantarana ny vola ao anatin'ny kaonty mTOMADY,, afaka ho resahinao amiko ve ny ho resahinao aminy?
4.5	Raha ny aminao manokana, inona ny fomba tena mety ahazoanao vaovao?<br><br>Afaka lazainao ve azafady aiza avy no afaka ahazoanao fanazavana fanampiny momba ny mTOMADY?
4.6	Inona ny fanazavana nirianao ho azo tany aloha momban'ny mTOMADY?<br><br>Fanadihadiana:<br>Ahoana ny fomba tianao nahafantarana ny mTOMADY?<br>Eny amin'ny toeram-pitsaboana, AC, video, aterineto, C-coach, olona eny amin'ny fokontany, peta-drindrina<br>Oviana no fotoana tena nety taminao ny nahafantatra ny fisian' ny mTOMADY?<br>Iza avy ny olona tokony horesahin'ny mTOMADY? Iza avy ao amin'ny tokantranonao? Iza ao anatin'ny fiaraha-monina?<br><br>Ahoana ny fomba ampahafantarana ihany koa ny…
4.7	Fanontaniana efatra sisa no apetrako aminao.<br><br>Ny tanjonay dia ny hahatonga ny mTOMADY hahasoa kokoa ny vehivavy. Inona no azontsika atao ho fanatsarana kokoa? Inona no mety ho fahadisoanay?<br><br>Fanadihadiana:<br><br>Inona no sakana amin'ny fisoratana anarana araka ny hevitrao?<br><br>Inona avy ireo sakana amin'ny fampiasana araka ny hevitrao?<br><br>Araka ny hevitrao, ny mTOMADY dia manampy kokoa ho an'iza, ary ho an'iza no tsy dia manampy izy?
4.8	MTOMADY dia miasa amin'ny alàlan'ny teknolojian'ny finday. Fantatsika fa mora kokoa ho an'ny olona sasany ny fampiasana izany, ary sarotra kokoa ho an'ny sasany. Ahoana ny hevitrao momba izany?<br><br>Fanadihadiana:<br><br>Ho anao, inona no mahamora ary inona no mahasarotra ny fampiasana vola amin'ny finday?<br><br>Inona no olana amin'ny fampiasana vola amin'ny finday?

4.9	Mba hazavao hoe azafady, inona no mahatonga anao hatoky na tsy hatoky ny mTOMADY.
4.10	*Iza no mahazo tombony betsaka indrindra amin'ny mTOMADY raha ny araka ny fijerinao?*<br><br>Fanadihadiana:<br><br>Iza no tsy mahazo tombony amin'ny mTOMADY?
4.11	Henonay hoe misy olona matahoatra mafy amin’ny mety hitranaga amin’ny volany. Ahoana ny eritreritrao amin’izany?<br><br>Fanadihadiana:<br><br>Raha ianao manokana, inona ny mety mampiahy anao amin’ny volanao? Henonay amin’ny olona sasany koa fa hoe mety ho mpisoloky ny mTOMADY. Inona ny azo antsoina hoe sandoka raha aminao manokana?

Fampahatsiahivana: Apetraho ny fanontaniana toy ny hoe: “Inona koa?” / “Misy zavatra hafa ve?”

LOHAHEVITRA 5: Ny fomba fijerin'ny fiaraha-monina<br>*Ireto fanontaniana irteo dia mikendry ny hahatakarana ny fandraisan'ny olona ny tolotra eo anivon'ny fiaraha-monina, izay tsy monina ao an-tokantranon'ilay mpamaly ny fanadihadiana*	
5.1	Farany, te-hahafantatra bebe kokoa momba ny hevitry ny olona samihafa momba ny mTOMADY izahay. Mba azonao hazavaina ve ny fomba iresahan'ny olona ao amin'ny fiaraha-monina misy anao momba izany?<br><br>Fanadihadiana:<br><br>Inona no nolazain'io olona io taminao?<br><br>Inona no andraikitr'io olona io eo amin'ny fiaraha-monina misy anao?<br><br>Inona avy ireo hevitra tena tsara eo amin'ny fiaraha-monina misy anao?<br><br>Inona avy ireo hevitra tena ratsy eo amin'ny fiaraha-monina misy anao?<br><br>Inona no teny ampiasain'ny olona ilazana ny mTOMADY?<br><br>Ahoana ny hevitrao momba azy ireo mampiasa ireo teny ireo?<br><br>Ahoana no niresahana izany fony ianao tany amin'ny tobim-pahasalamana?

	Efa re fa nisy tsaho momba ny mTOMADY tany amin'ny toerana sasany. Raha naheno momba izany ianao, azonao mba tanisaina ve?

LOHAHEVITRA 6: Famaranana ny tafatafa	
6.1	Misy zava-dehibe tsy mbola noresahintsika amin'ny fomba fijerinao ve? Misy zavatra tianao ampiana?
6.2	Misy zavatra tsy nanontaniako anao ve kanefa tokony hanontaniana anao?
6.3	Manana fanontaniana amiko ve ianao?

Misaotra anao indrindra amin'ny fandraisana anjara!

[Ajanona ny fandraisam-peo]

Fanadihadiana eny amin'ny fiaraha-monina
Mahafantatra olona hafa mety hahaliana amin(ity fanadihadiana ataonay ity ve ianao?
Mahafantatra olona nanana traikefa manokana (tsara na ratsy) tamin'ny MMHW ve ianao?
Miverina amin'ny fanontaniana hoe iza no nitaona anao: Heverinao ve fa afaka miresaka aminy [....] koa ve izahay?
Miverina amin'ny fanontaniana momba ny hevitra samihafa eo amin'ny fiaraha-monina misy anao: Heverinao ve fa mety ho afaka hiresaka aminy [....] koa ve izahay?
Afaka miverina aminao indray ve izahay mba hahafahanay mifandray amin'io olona io?

Semi-structured Interview guide – Household members

Thank you very much for agreeing to participate in another interview for our study on mTOMADY and the Mobile Maternal Health Wallet. Our aim in this part of the study is to understand why some people decide to use the intervention while others do not and especially how these decisions are made. Our goal is to improve the intervention to make it more accessible and beneficial.

I would now like to address a few general things about the project and the interview itself before we really get into it: First, as we described in the information sheet, participation is anonymous. This means that we make sure that all statements and information, that could enable somebody to identify you, are not linked to the answers you give in the interview. Participation is entirely voluntary. You can refuse to answer specific questions that you do not want to answer and you can stop the interview at any time. Please also let us know if you do not want to answer a specific question. To be able to properly analyse the interview later, we also need to record it. You can interrupt, restart or cancel the recording at any time. Therefore, you can use the buttons on the tablet. Do you have any further questions about it? Can I start the recording?

[START RECORDING]

Thank you! I would now like to talk with you about your experiences, perceptions and views on the Mobile Maternal Health Wallet including mTOMADY. There are no right and wrong answers, it is all about your personal experiences, views, and perceptions. We ask you to tell us everything you can think of.

**TOPIC 1:** "Ice-breaking question" and first thoughts<br><br>*This section should help to start a conversation, especially about the decision-making process.*	
1.1	First of all: How is your baby doing?
1.2	Please try to remember the first time somebody told you about mTOMADY. Explain the situation to me so I can feel a bit how you felt.<br><br>*Probes:*<br>Where were you?<br>When was it?<br>Who told you about it?<br>What were the first feelings and thoughts about it?<br>How did it go on afterwards?

Reminder: Ask probes like „Can you tell me more about this"? / "Then what happened?"

**TOPIC 2:** *Decision-making-process: personal perception, influences of household members and other people*<br><br>*The first questions focus on the personal perception of the decision-making process and main reasons, perceived advantages and disadvantages. Further, some questions aim to understand more about the women's environment and who influenced the decision.*	
2.1	*Doers:* Please try to remember how you thought about it and finally decided to register for mTOMADY. Please tell me the story of how the decision to register was taken.<br><br>*Non-doers:* We know that many people didn't sign up for mTomady. We can't talk to all of them but I can talk to you. Therefore, we would be happy if you could share some insights why you / your household didn't use the intervention: Please try to remember how you thought about it and finally decided not to register for mTOMADY. Please tell me the story of how the decision not to register was taken.
2.2	Who took the final decision?<br><br>*Probes:*<br>Please describe when the decision to use mTOMADY was taken during the pregnancy of your wife / girlfriend / daughter / ...?<br>Why did your household decide at this point of time?
2.3	Which reasons would you consider as the main ones for the decision?
2.4	What were the advantages for you?
2.5	What were the disadvantages for you?
2.6	Please explain how far your decision was influenced by members of your household if anybody had an influence.<br><br>*Probes:*<br>What was this person's opinion?<br>Why did this person's opinion influence your decision?<br>How large was this person's influence?<br>What would be the consequence if you would have decided against this person's

	opinion?<br>Who else in your household had an influence on the decision?<br>Who tried to convince you to use mTOMADY?<br>Was there anybody who told you not to use mTOMADY?
2.7	Please explain how far your decision was influenced by other people who are not members of your household.<br><br>*Probes:*<br>What was this person's opinion?<br>Why did this person's opinion influence your decision?<br>How large was this person's influence?<br>Who else had an influence on the decision?<br>Who tried to convince you to use mTOMADY?<br>Was there anybody who told you not to use mTOMADY?
2.8	We already heard that in some cases, there are community health workers that recommend mTOMADY and some not so much. How has the relation to a community health worker influenced your decision (not) to register?<br><br>*Probe:*<br>How is your relationship with the health worker in your community?<br>How good do you know the community health worker in your community?<br>How has the community health worker influenced your decision?<br>In which ways has the community health worker recommended mTOMADY or not?
2.9	If you could decide again now, how would you decide? And why?
2.10	What would have happened if your wife would register for mTomady even if you said that she shouldn't.

Reminder: Silence is a good probe

**TOPIC 3:** *Healthcare and healthcare expenses*<br><br>*These questions aim to help us to understand how the women we are interviewing are used to seeking healthcare, how they save for these purposes and if using mTOMADY changed something about this.*	
3.1	I would now like to talk to you about healthcare in general. Please think of a situation when one of your household members need to seek healthcare. Please explain the process that happens until you seek healthcare.

	Probes:<br>How does such a discussion start?<br>Who is generally involved in deciding whether one of your household members will seek healthcare?<br>Where would you usually seek care? Why would you seek care there?<br>If there are any, please tell me about other places where you would seek care.<br>Please describe factors that would keep your household from seeking care when you need it.<br>How do you take part in this process? (In which part?)
3.2	How would you describe the service your household receive for healthcare?<br><br>*Probes:*<br>How do you feel that your households needs are met?<br>What do you think of the time that is spent with your household?<br>How do you feel about the attention that is given to your household?<br>What do you think of the medication they give to your household?
3.3	How do you usually save for healthcare purposes if you do so?<br><br>*Probes:*<br>Which tools do you use for savings?<br>How often do you save?<br>For which situations do you save?
3.4	How have you been saving for the pregnancy of your wife / girlfriend / daughter / ... if you saved at all? Why did you do it like this?
3.5	*Doers only:* Getting back to mTOMADY: How did the way you save change by using mTOMADY if it changed?<br><br>*Probe:*<br>If it was not your first pregnancy, what made saving different from pregnancies before if it did at all?

Reminder: Probes can also be phrased like: "If you would compare x and y, what differences would you see?"

**TOPIC 4:** mTOMADY Barriers and improvements / Mobile Money<br><br>*These questions aim less on the decision-making but on general perception about the intervention, areas for improvement & trust in mTOMADY.*

4.1	*Doers:* mTOMADY was meant to help women to receive better care during pregnancy and childbirth. We know it's more helpful in some cases and in some less. How about you?<br><br>*Probes Doers:*<br>If you had children before: How was it different to have used mTOMADY compared to your pregnancies before if it was different at all?<br>How did you use mTOMADY?<br><br>*Non-doers:* mTOMADY was meant to help women to receive better care during pregnancy and childbirth. We know it's more helpful in some cases and in some less. If you had used mTomady, how do you think it would have been for you? Why? / why not?
4.2	How would you describe your feelings about mTOMADY over the time of the pregnancy of your wife / girlfriend / daughter / ...?<br><br>*Probes:*<br>Please explain if your feelings about mTomady changed.<br>What made these feelings change?<br>What was different between the beginning and the end of the pregnancy of your wife / girlfriend / daughter / ...?
4.3	*NOTE: Tell participant that there are 4 questions left.*<br><br>Our aim is to make mTOMADY more useful to women and households. What could we do better? How did we make mistakes?<br><br>*Probes:*<br>What are barriers for registration in your opinion?<br>What are barriers for usage in your opinion?<br>For whom do you think mTOMADY is more and for whom less helpful?<br>What additional information you would have liked to have but didn't receive?
4.4	mTOMADY works using mobile money technology. We know that some people find it easier, some more difficult to use. What do you think about it?<br><br>*Probes:*<br>What makes it easy and what makes it difficult for you to use mobile money?<br>What are problems with using mobile money?
4.5	Please explain what makes you trust or distrust mTOMADY.

4.6	Who profits most from mTOMADY from your point of view?<br><br>*Probe:*<br>Who doesn't profit from mTOMADY?

Reminder: Ask probes like: "What else?" / "Is there anything else?"

**TOPIC 5:** *Community perception*<br>*These questions aim to contribute to the understanding of how people in the community, who are not household members of the respondent, perceive the intervention.*	
5.1	Finally, we want to learn more about what different people think about mTOMADY. Would you please explain how people in your community talk about it?<br><br>Probes:<br>What did this person say to you?<br>What is this person's role in your community?<br>What are the very positive opinions in your community?<br>What are the very negative opinions in your community?<br>What words do people use to describe mTOMADY?<br>What do you think about them using these words?<br>How was this discussed when you were at the health centre?<br>We already heard that in some places there were rumours about mTOMADY. If you heard of any, would you please describe them?

**TOPIC 6:** End of the interview	
6.1	Is there anything important we have not talked about yet from your perspective? Anything you would like to add?
6.2	Is there anything I didn't ask you that I should have asked you?
6.3	Do you have any questions for me?

Thank you very much for participating!
[STOP RECORDING]

Interview guide for implementation team members:

Your daily work / Ny asanao andavanandro:

1. Please try to remember the start of the roll-out of MMHW. How was it getting started? How did you perceive it? Please tell us from the beginning to the end with as many details as possible.
   Ezaho tadidina ny fiantombohan'ny fametrahana ny MMHW. Ahoana no nanombohan'izany? Ahoana no fahatsapanao azy? Tantarao aminay hatramin'ny voalohany hatramin'ny farany amin'ny antsipirihany araka izay azo atao.
2. What was the sensitisation plan that you proposed to your team for this project? How did you find it? How did you change it?
   Inona ny drafitra fanentanana narosonao ho an'ny ekipanao momba ity tetikasa ity? Ahoana ny fahitanao azy io? Ahoana no nanovanao izany?
3. How did sensibilisation start? How was it going at the beginning? How did you feel about it? How did people react at the beginning?
   Ahoana no nanombohan'ny fanentanana ? Nanao ahoana ny fandehany tamin'ny voalohany ? Nanao ahoana ny fahatsapanao azy io? Nanao ahoana ny fihetsiky ny olona tamin'ny voalohany?
4. What challenges did you have at the beginning? What was done to solve these issues? Was there any particular thing you remember?
   Inona avy ireo fanamby napetrakao tany am-piandohana? Inona no natao mba hamahana ireo olana ireo? Nisy zavatra manokana tadidinao ve?
5. What changes were made over time?
   Inona ny fiovana natao rehefa nandeha ny fotoana?
   [ask for many details here]
   [Angataho antsipiriany maromaro eto]
   What led to these changes? What were the reasons?
   Inona no nahatonga izany fiovana izany? Inona no antony?
6. What are the main difficulties you have frequently?
   Inona ireo fahasarotana goavana hatrehinao matetika?
   What could be done to help you with this?
   Inona no azo atao hanampiana anao amin'zany?

Your approach to people:

Ny fomba fanatonanao ny olona:

1. What do you hear people saying about mT?

Inona no henonao resahan'ny olona mahakasika ny m-Tomady?

How is it perceived in general? Are there any very positive opinions? Are there very negative opinions? What rumors have you heard of?
Ahoana no fandraisan'ny olona azy amin'ny ankapobeny ? Misy hevitra tena tsara ve? Misy hevitra tena ratsy ve? Inona ny tsaho henonao?

2. How do you think the registration process could be improved?
   Araka ny hevitrao, ahoana no tokony hanatsarana ny dingana atao amin'ny fisoratana anarana?
   What prevents women from registering?
   Inona no manakana ny vehivavy tsy hisoratra anarana?
   Who do you think registers for MMHW and who doesn't?
   Araka ny hevitrao, iza no misoratra anarana amin'ny MMHW ary iza no tsy misoratra anarana?
3. What are the most common problems your team faces?
   Inona ireo olana mateti-pitranga hatrehan'ny ekipanao?
4. We heard that some people didn't hear about m-Tomady even if they went to facilities where it is used. How do you think more people could be reached to get information? how do you think more people could be reached to get registered?

   Renay fa misy olona sasany tsy naheno momba ny m-Tomady na dia nandeha teny amin'ny tobim-pahasalamana izay ampiasana azy aza izy ireo. Araka ny hevitrao, ahoana no ahafahana manatona olona bebe kokoa hahazoany fahalalana? Araka ny hevitrao, ahoana no ahafahana manatona olona bebe kokoa hitaomana azy hisoratra anarana?

   What ways of raising awareness are used currently?
   Inona ny fomba fanentanana ampiasaina amin'izao fotoana izao?
   What do you think can be done to improve the awareness about m-Tomady?
   Araka ny hevitrao, inona no azo atao hanatsarana ny fomba fanentanana momba ny m-Tomady?
   What should be done better to improve the situation?
   Inona no tokony atao hanatsarana izany toe-javatra izany?
5. We heard that other family members also have influence on the decision making process regarding m-Tomady. Who else do you think should be approached as well?

Renay fa misy fiantraikany eo amin'ny dingana fandraisana fanapahan-kevitra mikasika ny m-Tomady ireo olona ao anatin'ny fianakaviana. Araka ny hevitrao, iza koa no tokony hatonona?

What should be done better to improve the situation?

Inona no tokony atao hanatsarana izany toe-javatra izany?

6. We heard that some people get registered but have not clear information about m-Tomady use. Why do you think this happens? Many women said that they don't know how to deposit money themselves and don't know how to check their balance. What do you think about this?What should be done better to improve the situation?

   Renay fa misy olona sasany misoratra anarana kanefa tsy manana fahalalana mazava momba ny fampiasana ny m-Tomady. Araka ny hevitrao inona no mahatonga izany ? Maro ny vehivavy nilaza fa tsy mahay mametraka vola raha zareo ihany no manao azy ary tsy mahay mijery ny toe-bolany. Ahoana ny hevitrao momba izany ? Inona no tokony atao hanatsarana izany toe-javatra izany?

7. We heard that:

   Renay fa:

   People didn't get reimbursement

   Tsy naverina ny volan'ny olona

   People had to pay for registration (500- a lot)

   Tsy maintsy nandoa ny fisoratana anarana ny olona (500-betsaka)

   Some people thought mT would be a scam

   Nihevitra ny olona sasany fa fisolokina ny m-Tomady

   What do you think about this?

   Ahoana ny hevitrao momba izany?

   what should be done better to improve the situation?

   Inona no tokony atao hanatsarana izany toe-javatra izany?

hcp :

1. What do healthcare providers say to you about m-Tomady? What problems do they have?

Inona no lazain'ny mpiasan'ny fahasalamana momba ny m-Tomady? Inona ny olana hatrehany?

How could the work be eased for them?

Ahoana ny hanamaivanana ny asan'izy ireo ?

2. We heard that Some HCP Staff is not motivated about the intervention. Share with me if you have already experienced such a situation? And tell me what the main reason for that?

   Renay fa ny mpiasa sasany ao amin'ny HCP dia tsy nazoto hiditra an-tsehatra. Zarao amiko raha efa niaina toe-javatra toy izany ianao? Ary lazao ahy inona no tena antony?

   a. How did you know that they were not motivated about the intervention?

   a. Ahoana no nahafantaranao fa tsy nazoto hiditra an-tsehatra izy ireo?

   b. How did that influence your work?

   b. Inona no fiantraikan'izany teo amin'ny asanao?

3. We have heard that sometimes, HCP would keep the SIM Card after delivery.

   Renay fa indraindray ny HCP dia nitazona ny karatra sim taorian'ny fiterahana..

   a. Can you share your experience with such a situation if you have already been reported to such a situation?

      Afaka mba zarainao ve ny zavatra niainanao momba izany raha toa ka efa nolazaina toe-javatra toy izany ianao ?

4. Some women don't complain even when they think that something didn't work as they had expected. For e.g. when they didn't get their SIM Card back.

   Ny vehivavy sasany dia tsy mitaraina na dia mieritreritra aza izy ireo fa nisy zavatra tsy nety araka ny nantenainy. Ohatra, rehefa tsy naverina taminy ny karatra sim an'izy ireo.

   What do you think are the reasons for that?

   Araka ny hevitrao, inona no anton'izany ?

<u>other:</u>

<u>Hafa:</u>

1. What do you think could have been made to make m-Tomady better? What can be improved?

   Inona araka ny eritreritrao no azo atao hanatsarana ny m-Tomady? Inona no azo hatsaraina?

2. What was the biggest frustration that you encountered in your work?

   Inona no zavatra nanafintohina anao indrindra natrehanao teo amin'ny asanao ?

3. How would you describe the technical support you received from the team?
   Ahoana no hamaritanao ny fanohanana ara-teknika azonao avy amin'ny ekipa?
