# Supplementary figures and images for: What compels enrollment in a mobile maternal health wallet? A mixed-methods doer/non-doer analysis in Analamanga, Madagascar
Source: BMC Health Serv Res. 2025 Dec 6;25:1584. doi: 10.1186/s12913-025-13770-x (PMC12687524; doi:10.1186/s12913-025-13770-x)

Supplementary file 3: Flowchart: Lost due to missing information for the quantitative analysis

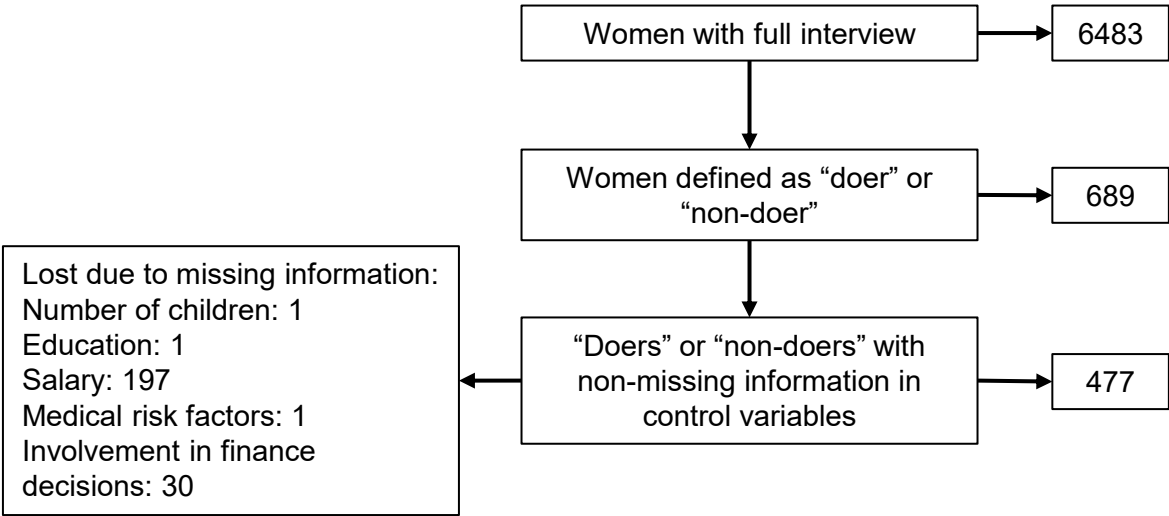

Supplement: Supplementary file 3 — Supplementary Material 3 [file 12913_2025_13770_MOESM3_ESM.pdf]
