# Supplementary material for: What compels enrollment in a mobile maternal health wallet? A mixed-methods doer/non-doer analysis in Analamanga, Madagascar
Source: BMC Health Serv Res. 2025 Dec 6;25:1584. doi: 10.1186/s12913-025-13770-x (PMC12687524; doi:10.1186/s12913-025-13770-x)
Supplement: Supplementary file 4 — Supplementary Material 4 [file 12913_2025_13770_MOESM4_ESM.pdf]

Supplementary file 4: Original qualitative quotes in Malagasy

Interview	Malagasy	English
Main text		
DND29	„Ohatry ny nandehandeha ho azy teny fotsiny ny fiainako dia tsy niditra aho“	“My life seemed to be just going well, and I did not enroll”
DND26	„Ny nahatonga ahy natoky ny m-Tomady dia nahita ilay rahavaviko sy ilay tompon-tranonay taloha nanao azy dia tena nety tamin-dry zareo ilay izy. […]“	“What made me trust the MMHW was seeing my sister and our previous landlord using it, it served them very well […]”
DND13	“[…] vao azon'ny mTOMADY ilay volan'olona dia blokeny ilay puce”	“[…] take their money and block the SIM card afterwards[…]”
DND03	“Tsy naharisika ahy kosa aloha ilay izy e satria efa nahare feo aho hoe ilay mTOMADY magalatra […] Taorinan'izay dia rehefa bevohoka moa aho dia izay aho vao tena nirisika hoe aleo aho hanao satria ilay mihaino vavan'olona koa tsy mety“	“[The MMHW] didn’t impress me, because I heard [the platform] would steal […]. Later, when I was pregnant, I took the risk and thought we would see, because just listening to what someone‘s saying is not good!”
DND11	“Ilay fisian'ny echo tamin'ny mTOMADY no nampiavaka azy fa ilay taloha miandry ho teraka dia teraka. Niaraka tamin'ny mTOMADY mba efa afaka nivonona hoe ‘izao zanako ity e, ho teraka soa aman-tsara zanako ity e’”	“Ultrasounds […] make a difference. In the past you waited for the birth and that is all. With the MMHW you are able to say: ‘This is my baby; it is going to be born safely’”
DND18	„Izy indray aloha raha vola tsy tianao ho lany loatra dia aleo apetraka any anaty telefonina! Fa raha mipetraka ao an-trano izy dia tsy maintsy voasarikao. Sahala amiko manokana izao raha misy manome vola aho ka mbola tsy dia tena ilaiko dia apetrako any anaty telefonina“	"If it is money that you don’t want to spend frivolously, you should put it in the phone. If you keep it at home, you are inevitably going to use it. Personally, if someone gives me money and I do not really need it, I put it in my phone."
DND04	Interviewer: „Dia rehefa misy tsy fahasalamana dia ahoana ny fiaingan'ny dinika ato aminareo ato na-tokantrano, iza no mandray anjara amin'ilay resaka?“<br>Respondent: „Izaho“<br>Interviewer: “Ianao? Ary miresaka amin'ny vadinao ve ianao? Miara-miresaka ve nareo?“<br>Respondent: „Miresaka izahay fa izaho foana no tompony fanapahakevitra“	Interviewer: “When there is a sick person at home, how does the discussion start, who is<br>involved?”<br>Respondent: “Me”<br>Interviewer: “You? And you talk with your husband? Do you talk together?”<br>Respondent: “We discuss but I make the decision.”
Table 3		
DND09	Teny amin'ny CSBII […]. Teny no nisy mpanentana nitantara hoe ohatr'izao ny mTOMADY, afaka manampy anareo. Raha misy ny probleme eo am-piterahana dia afaka manampy anareo ny mTOMADY. Indrindra indrindra amin'ny resa-bola. Dia izahay moa ao	“It was at the CSB […]. There, an MMHW agent explained what the MMHW is and said that it can help you. If you have a problem during childbirth, the MMHW can help you. Especially with the money. As we are in need, we were very motivated,

	anatin'ny fahasahiranana dia tena narisika tanteraka satria sao dia hoe misy fahasarotana izany dia efa miantoka ny mTOMADY. Izay no tena naharesy lahatra ahy tamin'ilay izy izany	because in case of complications, the MMHW would help.”
DND02	Izaho mantsy tamin'iny izy mantsy niteny hoe manontania manana fanontaniana dia izaho saika hanontany azy raha tsy manambola mihitsy izany ampidirina ao dia ahoana [mihomehy] Dia tsy nanontany azy aho fa izaho irery koa ve no anontany eo dia tsy nisy nanontany izahay rehetra avy teo.	“[The MMHW agent] told me to ask questions if I had any and my question was: ‘If I don’t have any money to put in the SIM-card [laughs], what will happen?’ And I didn’t ask the question because it was only me who had the question and the others didn’t ask as well!”
DND10	Tsy dia hotako izay tombony satria mitovy ihany. Samy vola ho aloha ihany. Tsy hitako hoe inona no fatiantoka amin'ilay izy fa mitova ihany na niditra na tsy niditra.	“I don't see the advantage because it's the same. We always must pay money. […] I do not see any disadvantage; it is the same whether we enroll or not.”
DND02	Izaho aloha izany tsy nanana eritreritra hiditra tamin'ilay izy satria ny antony? Za mantsy a ataoko efa roa volana alohan'ny tsy ahaterahako aho vao nahita anazy satria izahay moa misafo eny foana fa indrindray izy tsy eny an dia indraindray izy […] Dia tsy rototra intsona aho tamin'ilay izy satria ozy aho hoe he he he izaho efa ho teraka dia tsy dia ilaiko loatra ilay izy.	“First, I didn’t intend to enroll. Why? Because two months before I gave birth, I met [the MMHW agent]! Because I usually did the prenatal examination [in a CSB] and sometimes [the agent] was there and sometimes not […] And I wasn’t interested anymore because no... I thought I ‘m already about to give birth and I don’t really need it!”
DND11	Mizara roa aloha ilay firesaky ny fiarahamonina e. Ny sasany izany […] milaza hoe aza manao an'io intsony fa tsy misy dikany. Ny sasany kosa anefa mampirisika hoe manaova mTOMADY satria tena maivana tamin'ny hono ny fiterahana, tsy nandany vola be izy.	“The way they talk is divided in two. Some […] say don't enroll because it does not make sense. The others encourage you to use the MMHW because as they used it, the delivery was lighter for them. They did not spend a lot of money.”
DND09	Izaho ary mahita an'ilay ankizy bevohoka sasany ety dia miezaka miteny amin-dry zareo hoe manaova mTOMADY fa hafa mihitsy ny ao anatin'io. […] Izaho manazava amin-dry zareo ety hoe ohatr'izao no ao anatin'ny mTOMADY.	“When I meet some of the pregnant women here, I advise them to [enroll in the MMHW] because it is really great to be a member. [,,,] I explain to them what we can get from the MMHW.”
DND14	Tena tsy mitahiry mihitsy izahay satria olona sahirana. Raha misy ny tampoka dia tsy maintsy mindrana. Satria izay tonga lany sady tsy mety raha ho eny hohanina aza tsy misy kanefa ahitsoka ao fotsiny ilay vola.	“We never save because we are in need. If there is an emergency, we have to borrow. […]. Because you spend what you earn and you cannot block the money there when you have nothing to eat.”
DND23	Raha amiko manokana dia voalohany indrindra ilay resaka mpisoloky ohatra. Ilay resaka mandoha vola mihitsy no nahatonga sainy ahy hoe iza no handohavana vola, aiza, dia iza no	“Personally, first of all the question of scamming for example. The question of payment made me think: Who are you going to give the money to? Where? Who is going to check it? […] it was just a

	manamarina ilay izy. […] fa tamin'ireny resadresaka rasazy teny an-dalana fotsiny. Efa betsaka ny mpisoloky no tena marina.	conversation in the street with the midwife. The truth is that there are many scammers."
DND29	Interviewer: Iza no miara-miresaka an'izay?<br>Respondent: Izahay mivady ihany<br>Interviewer: Iza no tena manapa-kevitra hitady fitsaboana?<br>Respondent: Miara-manapaka.	Interviewer: "Who discusses [health issues in the family]?"<br>Respondent: "My husband and I."<br>Interviewer: "And who decides to seek treatment?"<br>Respondent: "We decide together."
DND19	Izay efa noteneniko izay hoe ny izahay mivady no miresaka fa izy no tena manapa-kevitra matetika	"As I said, it is me and my husband who discuss but he often decides."
DND27	Ny eritreritro hoe te hiditra amin'ilay izy aho fa mbola sahirana tamin'ilay izaho bevohoka dia ilay izy koa mbola nisy etape fidirana.	"I thought about enrolling but I was still struggling because I was pregnant and then there were several steps to enroll."
DND07	Ie, ilay izy aloha moramora. Ilay mampiasa an'ilay telefonina. Fa ilay sakany misy ilay sasany tsy mahay mampiasa ilay telefonina satria rehefa tsy mahay ianao dia tsy maintsy mankany amin'ny CSBII manao an'iny	"Yes, using the phone is quite easy. The obstacle that exists is that some people don't know how to handle a phone and would have to go to a CSB to do so."
DND18	Nanatona an-dry zareo tao izahay satria vonona ny anao an'ilay izy dia nitondra ny karapanondrony tany izy. Isaky ny mandeha misafo izy na tsy misafo koa aza dia manatitra vola any. Na tsy vola be aza moa dia azo arotsaka hoy ilay olona tao, na dia 1000ariary aza dia efa mety. Tena nisy tombony be ilay izy satria tonga tokoa ilay andro iray kanefa izahay tao anatin'ny tsy fisiana. Ilay puce sy karapanondrony no nentina, izy koa moa teny amin'ny CSBII […] ihany no niteraka, dia iny no nandohavana ny vola lany tao.	"We approached [the MMHW agent] because my daughter was ready to [enroll], she brought her ID card there. Whether or not she did her prenatal consultation, she came there to deposit money. Even if it is not a lot of money, they said, you could always deposit even 1,000 ariary. There is a great advantage because when the day arrived, we were in a period of nothing. We only took the SIM card and the identity card with us, she gave birth at the CSB […], and we paid the expenses with [the MMHW]."
