# Supplementary material for: What compels enrollment in a mobile maternal health wallet? A mixed-methods doer/non-doer analysis in Analamanga, Madagascar
Source: BMC Health Serv Res. 2025 Dec 6;25:1584. doi: 10.1186/s12913-025-13770-x (PMC12687524; doi:10.1186/s12913-025-13770-x)
Supplement: Supplementary file 5 — Supplementary Material 5 [file 12913_2025_13770_MOESM5_ESM.docx]

Supplementary file 5: French translation of the abstract

RESUMÉ : Qu’est-ce qui incite à s’inscrire dans un portefeuille mobile de santé maternelle ? Une analyse par méthodes mixtes des *« doers » et « non-doers »* à Analamanga, Madagascar

Auteurs : Louis Noël Schäfer, Zavaniarivo Rampanjato, Lisa Bogler, Elsa Rajemison, Samuel Knauss, Julius Valentin Emmrich, Mirana Larissa Randriamiarisoa, Harizaka Emmanuel Andriamasy, Louisa Marie Truss, Bítia Vieira, Till Bärnighausen, Mark Donald C. Reñosa, Shannon A. McMahon

Full article available in English - DOI: 10.1186/s12913-025-13770-x

**Contexte :** Le recours aux soins pendant les périodes prénatale et intrapartum reste faible à Madagascar, en grande partie en raison d'obstacles financiers. Afin de renforcer à la fois le recours aux soins et le financement des soins de santé, un portefeuille mobile pour la santé maternelle (Mobile Maternal Health Wallet, MMHW) a été développé et mis en œuvre dans la région d'Analamanga, Madagascar. Le MMHW, un service basé sur l'argent mobile, permettait aux utilisatrices d’épargner et de payer numériquement les services dans les établissements publics de santé participants. Nous comparons ici les points de vue des femmes qui se sont inscrites (« *doers »*) et de celles qui ne se sont pas inscrites (« *non-doers »*) au MMHW afin de comprendre les processus décisionnels, les dynamiques interpersonnelles et d’autres facteurs ayant influencé l'inscription.

**Méthodes :** Dans cette étude utilisant des méthodes mixtes, nous avons analysé les données d'une enquête quantitative (n = 477) examinant les facteurs prédictifs de l'inscription à l'aide de modèles de régression logistique, puis nous avons appliqué une analyse thématique réflexive (« *Reflexive Thematic Analysis* ») à des entretiens qualitatifs approfondis (n = 29) afin d'obtenir des informations auprès de 11 « *doers* », 12 « *non-doers* », trois membres des familles de « *doers* » et de « *non-doers* », et trois membres de l'équipe de sensibilisation du MMHW.

**Résultats** : Concernant les données quantitatives, les facteurs prédictifs significatifs de l'inscription comprenaient notamment le fait d’avoir été informée du MMHW par une sage-femme, de présenter un facteur de risque médical maternel préexistant et de résider dans le district d'Avaradrano à Antananarivo. Les facteurs prédictifs de non-inscription comprenaient le fait d’avoir été informée du MMHW par la famille ou des amis, d’avoir un salaire plus élevé et d’avoir reçu un diagnostic de signes avant-coureurs médicaux pendant la grossesse. Les résultats qualitatifs reflétaient en grande partie les données quantitatives, révélant que les femmes s'étaient inscrites grâce à des informations complètes fournies à un stade précoce de leur grossesse par des sources fiables (par exemple, des sage-femmes) et parce qu'elles trouvaient l'offre d'avantages financiers et de services médicaux élargis pertinente et crédible. Les femmes ont parfois indiqué que l'influence de leurs pairs avait joué un rôle dans leur décision de s'inscrire, mais pas systématiquement. Les femmes « *non-doers* » ont décrit un sentiment de méfiance à l'égard du MMHW (citant souvent des rumeurs ou les expériences négatives d'autres personnes), des informations incomplètes, des désaccords avec leur conjoint au sujet de l'inscription et des obstacles liés à la mise en œuvre du MMHW (par exemple, l’absence de carte d'identité, nécessaire à l'inscription).

**Conclusion** : Cette étude souligne l'importance d'évaluer les activités de sensibilisation auprès des populations cibles. Nos résultats mettent en évidence un besoin d'identifier des sources d'information fiables, qu’il s’agisse de personnes ou de canaux de communication, et de diffuser des programmes innovants par l'intermédiaire de ces sources. Une communication efficace et l'élimination des obstacles à la mise en œuvre restent essentielles pour améliorer le recours aux soins et les résultats en matière de santé maternelle à Madagascar et au-delà.

**Enregistrement de l'essai :** Cette étude fait partie de l'essai 4MOTHERS, qui a été enregistré le 12 mars 2021 dans le registre allemand des essais cliniques (DRKS), sous le numéro DRKS00014928, <https://drks.de/search/en/trial/DRKS00014928>.
